# A Comprehensive Review of Magnetic Nanocatalysts for C−S, C−Se Bond Formation

**DOI:** 10.1002/open.202500041

**Published:** 2025-03-27

**Authors:** Radwan Ali, Jianlin Han, Mosstafa Kazemi, Ramin Javahershenas

**Affiliations:** ^1^ Al-Qadisiyah University College of Dentistry Department of Basic Sciences, Qadisiyyah, Iraq.; ^2^ Jiangsu Co-Innovation Center of Efficient Processing and Utilization of Forest Resources College of Chemical Engineering Nanjing Forestry University Nanjing 210037 China; ^3^ Young Researchers and Elite Club Tehran Branch Islamic Azad University Tehran Iran; ^4^ Department of Organic Chemistry Faculty of Chemistry Urmia University Urmia Iran

**Keywords:** magnetic nanocatalysts, C−S bond, C−Se bond, Synthesis, heterocycles

## Abstract

This review manuscript examines magnetic nanocatalysts and their pivotal role in forming carbon‐sulfur (C−S) and carbon‐selenium (C−Se) bonds. The study delves into the latest advancements in the synthesis, characterization, and application of magnetic nanocatalysts, highlighting their unique advantages, including enhanced catalytic activity, superior selectivity, and easy recovery through magnetic separation, which align with the principles of green chemistry. Through a critical analysis of recent research findings, this review also explores the mechanistic pathways facilitated by these nanocatalysts, offering insights into their operational efficiency and potential for recyclability. The manuscript aims not only to catalog the current achievements in this burgeoning field but also to identify challenges and propose future directions for developing more efficient, sustainable, and versatile catalytic systems for C−S and C−Se bond formation. By encompassing a broad spectrum of magnetic nanocatalysts, ranging from bare magnets to functionalized and composite materials, this review is a comprehensive resource for researchers engaged in organic synthesis, catalysis, and sustainable chemistry.

## Graphical abstract

This review manuscript provides an exhaustive examination of magnetic nanocatalysts and their pivotal role in the formation of carbon‐sulfur (C−S) and carbon‐selenium (C−Se) bonds. The study delves into the latest advancements in the synthesis, characterization, and application of magnetic nanocatalysts, highlighting their unique advantages, including enhanced catalytic activity, superior selectivity, and easy recovery through magnetic separation, which align with the principles of green chemistry.

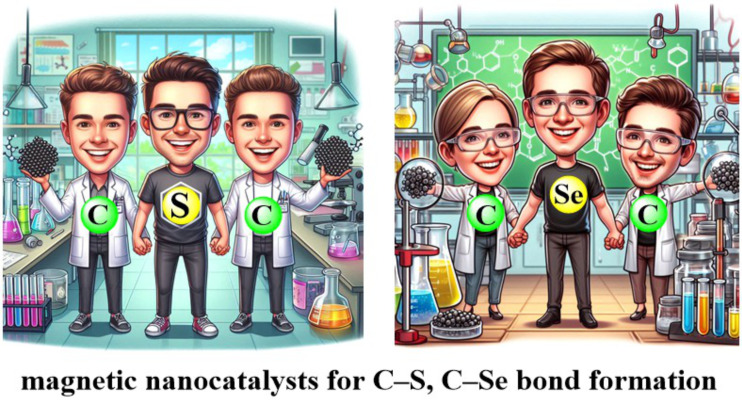

## 1 Introduction

Recent advancements in forming carbon‐sulfur (C−S) and carbon‐selenium (C−Se) bonds have focused on more sustainable and efficient methods. A notable development for C−S bond formation is the use of photocatalyzed reactions, which allow for the synthesis of thioesters from feedstock chemicals and elemental sulfur. Forming carbon‐sulfur (C−S) and carbon‐selenium (C−Se) bonds is fundamental in organic synthesis and has significant implications across various fields, including pharmaceuticals, agrochemicals, and materials science. Compounds containing C−S and C−Se bonds exhibit various biological activities and unique material properties, making them indispensable in developing drugs, crop protection agents, and functional materials. Traditional methods for synthesizing these bonds often involve harsh reaction conditions, toxic reagents, and complex purification steps, which limit their practicality and environmental friendliness.[^1^, ^2^, ^3^, ^4^, ^5^]

In recent years, magnetic nanocatalysts have emerged as a powerful class of catalysts that offer remarkable advantages in synthesizing C−S and C−Se bonds. These nanocatalysts combine the unique properties of nanomaterials—such as high surface area, enhanced reactivity, and tunable surface chemistry—with the benefits of magnetic properties, which facilitate easy separation and recovery from reaction mixtures. Using magnetic nanocatalysts aligns with the principles of green chemistry, aiming to reduce waste, minimize environmental impact, and improve the overall efficiency of chemical processes. Magnetic nanocatalysts such as copper complexes immobilized on magnetic nanoparticles have been developed for C‐ S bond formation. These catalysts are efficient, eco‐friendly, and can be quickly recovered and reused, aligning with green chemistry principles.

Additionally, Fe_3_O_4_‐based nanocatalysts have been used for selective oxidation processes, demonstrating high catalytic activity and reusability. Iron‐catalyzed reactions have been explored for C(sp^3^)‐Se bond cross‐coupling in the context of C‐ Se bond formation, providing an economical and efficient approach. These advancements highlight the potential of magnetic nanocatalysts to enhance the efficiency and sustainability of chemical processes involving C−S and C−Se bonds, making them valuable tools in organic synthesis.[^6^, ^7^, ^8^, ^9^, ^10^]

The use of magnetic nanocatalysts for C−S and C−Se bond formation is an emerging area of research with significant potential. These catalysts leverage nanomaterials′ high surface area and reactivity, combined with magnetic properties for easy separation and recovery.

This comprehensive review aims to systematically analyze the current research on magnetic nanocatalysts specifically designed for C−S and C−Se bond formation. We will explore various synthetic strategies employed to develop these nanocatalysts, their mechanistic insights, and the diverse range of substrates they can effectively transform. Recent studies have explored various synthetic strategies for developing these nanocatalysts. For instance, iron and nickel salts have been used to catalyze selective C−S and C−Se bond formation through C−H activation, demonstrating the ability to tolerate various functional groups.[^11^, ^12^, ^13^, ^14^, ^15^]

Additionally, we will highlight the advancements in characterization techniques that have facilitated understanding these materials at the nanoscale, providing insights into their catalytic performance. By consolidating existing knowledge and identifying gaps in the literature, this review seeks to provide a valuable resource for researchers aiming to advance the field of magnetic nanocatalysis and broaden its application in organic synthesis. Mechanistic insights into these processes reveal the role of specific ligands and reaction conditions in enhancing catalytic performance. For example, iron‐catalyzed oxidative C−H functionalization has been shown to facilitate C−S bond formation under mild conditions, with pyridine playing a crucial role in achieving high yields and selectivities.[^16^, ^17^, ^18^, ^19^, ^20^]

A critical analysis of recent literature highlights the successes and challenges associated with magnetic nanocatalysts, including catalyst stability, reusability, and scalability issues. By examining these aspects, the review identifies potential areas for future research and development, proposing strategies to overcome current limitations and enhance the applicability of magnetic nanocatalysts in industrial settings. Recent literature highlights both successes and challenges associated with magnetic nanocatalysts in forming C−S and C−Se bonds. These catalysts offer significant advantages, such as enhanced reactivity and easy recovery due to their magnetic properties, which align with green chemistry principles. However, challenges remain regarding catalyst stability, reusability, and scalability. For instance, while copper catalysts are less toxic and more economical than palladium, they still face issues related to catalyst poisoning and harsh reaction conditions. The stability and reusability of these catalysts are critical for their practical application, as they must maintain activity over multiple cycles without significant degradation. Scalability is another challenge, as processes that work well on a small scale may not translate effectively to industrial settings.[^21^, ^22^, ^23^, ^24^, ^25^]

In summary, magnetic nanocatalysts represent a promising and evolving area in synthesizing C−S and C−Se bonds, offering significant improvements in sustainability, efficiency, and practicality. This review provides valuable insights into the current state of the art and future directions for researchers and practitioners in the field, highlighting the potential of magnetic nanocatalysts to revolutionize C−S and C−Se bond formation and contribute to greener and more sustainable chemical processes.

## 2 C−S and C−Se Bond Formation in Organic Synthesis

The formation of carbon‐sulfur (C−S) and carbon‐selenium (C−Se) bonds is a critical area in organic synthesis, with various methods and mechanisms explored in recent literature. Compounds featuring C−S and C−Se bonds are present in various pharmacologically active agents, agrochemicals, and functional materials. This overview delves into the methods and mechanisms of C−S and C−Se bond formation, shedding light on traditional and state‐of‐the‐art approaches in this essential domain of organic chemistry.[^6^, ^7^, ^8^, ^9^, ^26^, ^27^, ^28^, ^29^, ^30^]

### 2.1 C−S Bond Formation

1. **Traditional Methods**: Early methods often involved the condensation of metal thiolates with organic halides. However, these methods were limited by harsh conditions and the limited substrate scope.
2. **Modern Approaches**: Recent advancements include copper‐catalyzed reactions using Na_2_S_2_O_3_ as a sulfurating agent, which are mild and compatible with biomolecules. Radical‐radical cross‐coupling has also been explored for asymmetric diaryl thioethers.

### 2.2 C−Se Bond Formation

1. **Transition‐Metal‐Free Methods**: Organoboron compounds have been used for transition‐metal‐free C−Se bond formation, offering a greener alternative with high molecular diversity.
2. **Oxidative Functionalization**: Direct oxidative functionalization of C(sp_3_)‐H bonds with diselenides provides a simple and atom‐economical method for selenide synthesis.

The significance of C−S and C−Se bonds in pharmaceuticals, agrochemicals, and materials science is well‐documented due to their diverse biological activities and functional properties. Sulfur‐containing compounds, such as thioethers, thiophenes, and sulfonamides, exhibit various biological activities, including antimicrobial, antifungal, and anticancer properties. Selenium‐containing compounds, on the other hand, are recognized for their roles as antioxidants and chemopreventive agents. Their ability to participate in redox reactions makes C−Se bonds critical in developing therapeutics to modulate oxidative stress and related conditions.[^31^, ^32^, ^33^, ^34^, ^35^]

C−S and C−Se bonds are crucial for developing drugs with diverse therapeutic applications in the pharmaceutical industry. Sulfur‐containing compounds, such as thioethers, thiophenes, sulfoxides, and sulfonamides, are present in myriad pharmaceutical agents with activities ranging from antimicrobial and antifungal to anticancer and anti‐inflammatory. For instance, the presence of sulfur in penicillin and cephalosporin antibiotics is key to their ability to inhibit bacterial cell wall synthesis. Similarly, selenium‐containing compounds are vital due to their antioxidant properties, which are leveraged in drugs to reduce oxidative stress and manage related diseases. The biochemical versatility provided by these bonds is enhanced by their ability to modulate the lipophilicity, electronic properties, and metabolic stability of drug molecules. This modulation is crucial for optimizing drug absorption, distribution, metabolism, and excretion (ADME) profiles, ultimately improving pharmaceutical agents′ therapeutic efficacy and safety.[^36^, ^37^, ^38^, ^39^, ^40^, ^41^, ^42^, ^43^, ^44^, ^45^]

In agrochemicals, C−S and C−Se bonds are essential for the functionality of pesticides, herbicides, and fungicides. Sulfur‐containing agrochemicals, such as thiocarbamates and dithiocarbamates, effectively control various pests and diseases, safeguarding crop yields and quality. Including sulfur imparts these compounds with broad‐spectrum activity and stability under various environmental conditions. For example, the herbicide S‐metolachlor, which contains a C−S bond, is widely used for controlling grasses and broadleaf weeds. Selenium, though used to a lesser extent, plays an important role in certain agrochemicals where its redox activity can be harnessed for plant protection and growth regulation. Compounds with C−Se bonds have been explored for their ability to interact with biological systems in ways that enhance plant resilience to biotic and abiotic stresses.[^46^, ^47^, ^48^, ^49^, ^50^]

In materials science, incorporating sulfur and selenium into polymer backbones and other advanced materials provides unique physical properties exploited in various high‐performance applications. Sulfur‐containing polymers, such as polysulfides and thiophene‐based conjugated polymers, exhibit enhanced thermal stability, chemical resistance, and electrical conductivity, making them suitable for applications in batteries, sensors, and organic electronics. Selenium, with its semi‐conductive properties, is pivotal in developing materials for photovoltaic cells and other optoelectronic devices. Its ability to form stable C−Se bonds with carbon‐based frameworks allows for designing materials with tailored optical and electronic properties, enabling advancements in next‐generation solar energy conversion and photodetector technologies.[^51^, ^52^, ^53^, ^54^, ^55^]

## 3 Magnetic Nanocatalysts

Magnetic nanocatalysts represent an innovative and rapidly advancing field in catalysis, characterized by integrating catalytic properties with magnetic behavior at the nanoscale. These materials have garnered significant attention due to their unique ability to combine high catalytic efficiency with easy recoverability, contributing substantially to the principles of green chemistry and sustainable industrial processes. Magnetic nanocatalysts typically consist of magnetic nanoparticles (MNPs) such as iron oxide (Fe3O4 or Fe2O3), cobalt ferrite, or nickel ferrite, stabilized by various coatings or functionalized surfaces. The magnetic core provides inherent magnetic properties, enabling catalysts to be separated from reaction mixtures using an external magnetic field. This simplifies the recovery and reuse of the catalyst, reducing waste and minimizing the use of additional separation processes.[^56^, ^57^, ^58^, ^59^, ^60^]

The core magnetic nanoparticles are often coated or functionalized with metal catalysts, organic ligands, or other catalytic entities to enhance their activity and selectivity. The coating also protects the magnetic core from agglomeration and environmental degradation, extending the catalyst‘s lifespan and maintaining its efficiency over multiple reaction cycles. Magnetic nanocatalysts demonstrate exceptional catalytic activity due to the high surface area‐to‐volume ratio characteristic of nanoparticles. This large surface area provides abundant active sites for reactant molecules, facilitating more efficient interactions and accelerating reaction rates. Furthermore, the surface of magnetic nanocatalysts can be precisely engineered to promote specific reactions, thereby improving selectivity and yield.[^61^, ^62^]

Magnetic nanocatalysts have gained significant attention due to their unique properties and applications in various catalytic processes. These nanocatalysts, often composed of iron oxides or ferrites, offer advantages such as high surface area, ease of recovery, and reusability, making them suitable for various organic transformations and biodiesel production. The following sections outline the primary types of magnetic nanocatalysts and their applications (Table 1).[^63^, ^64^, ^65^, ^66^, ^67^, ^68^, ^69^, ^70^, ^71^, ^72^, ^73^, ^74^, ^75^, ^76^, ^77^]

**Table 1:** Types of Magnetic Nanocatalysts.

Type of Magnetic Nanocatalyst	Description	Examples
Superparamagnetic Nanoparticles	Exhibit magnetic behavior only in the presence of an external magnetic field; do not retain magnetization when the field is removed.	Iron oxide nanoparticles (Fe_3_O_4_, γ‐Fe_2_O_3_)
Ferromagnetic Nanoparticles	Retain magnetization even after the external magnetic field is removed; typically larger in size.	Cobalt ferrite (CoFe_2_O_4_)
Metal‐Based Magnetic Nanoparticles	Composed of magnetic metals or metal oxides, providing strong magnetic properties and catalytic activity.	Iron oxide (Fe_3_O_4_), Cobalt oxide (Co_3_O_4_)
Composite Magnetic Nanoparticles	Consist of a magnetic core and a catalytic shell, combining the advantages of both components for enhanced performance.	Magnetic core‐shell nanoparticles
Magnetic Polymer Nanoparticles	Incorporate magnetic materials within a polymer matrix, offering flexibility and functionalization options.	Polymeric nanoparticles with iron oxide

Magnetic nanocatalysts have gained prominence in various catalytic processes, particularly biodiesel production, due to their unique properties such as high surface area, ease of separation, and reusability. The synthesis methods for these nanocatalysts are crucial as they directly influence their catalytic performance and stability. Below are the common synthetic methods for magnetic nanocatalysts (Table 2).[^78^, ^79^, ^80^, ^81^, ^82^, ^83^, ^84^, ^85^, ^86^, ^87^, ^88^, ^89^, ^90^]

**Table 2:** Overview of Common Synthetic Methods for Magnetic Nanocatalysts.

Synthetic Method	Description	Advantages	Disadvantages
Co‐precipitation	Mixing of metal salts and base in aqueous solution to precipitate magnetic nanoparticles.	Simple, scalable, cost‐effective	Particle size distribution may be broad
Thermal Decomposition	Decomposing organometallic compounds at high temperatures in the presence of surfactants.	Precise control over particle size and shape	Requires high temperatures, potentially hazardous
Hydrothermal/Solvothermal Synthesis	Using high temperature and pressure in water or organic solvents to crystallize nanoparticles.	Uniform particle size, high crystallinity	Requires autoclaves, longer reaction times
Microwave‐Assisted Synthesis	Using microwave irradiation to heat reactants rapidly and uniformly for nanoparticle formation.	Rapid synthesis, energy‐efficient	Limited to specific microwave‐compatible setups
Sol‐Gel Method	Hydrolysis and condensation of metal alkoxides forming a gel, which is then calcined to obtain nanoparticles.	High purity products, low‐temperature processing	Time‐consuming, may require post‐synthesis modifications
Sonochemical Synthesis	Using ultrasonic waves to induce cavitation and produce nanoparticles.	Low reaction temperatures, fine particle formation	Limited scalability, possible equipment damage
Polyol Method	Utilizing polyols as solvent and reducing agent to synthesize nanoparticles.	High boiling points allow fine control of conditions	High viscosity could complicate purification processes
Reverse Micelle Synthesis	Using surfactants to form micelles that control the nucleation and growth of nanoparticles.	Nanosize control, high uniformity	Use of surfactants can complicate purification
Electrochemical Synthesis	Electrolysis of metal salts in the presence of a stabilizing agent to form nanoparticles.	Fine control over particle composition and size	Requires electrical setup, limited scalability
Template‐Assisted Synthesis	Use of templates to guide the formation and morphology of nanoparticles.	Control over shape and size, high uniformity	Template removal can be complex and costly

Magnetic nanocatalysts offer a compelling solution to these challenges by combining the high catalytic activity and selectivity of nanomaterials with the ease of magnetic separation.[^91^, ^92^, ^93^, ^94^, ^95^, ^96^, ^97^, ^98^, ^99^, ^100^] This unique combination addresses several key issues in Table 3:

**Table 3:** Advantages and disadvantages of Magnetic Nanocatalysts.

Advantages of Magnetic Nanocatalysts		Disadvantages of Magnetic Nanocatalysts	
Easy Recovery	MNCs can be easily separated from reaction mixtures using an external magnetic field, minimizing product loss and facilitating catalyst recycling.	Scalability Challenges	While effective at the laboratory scale, scalability of magnetic nanocatalysts to industrial levels can be challenging due to synthesis and functionalization complexities.
High Catalytic Efficiency	The high surface area of magnetic nanoparticles increases the availability of active sites, leading to enhanced reaction rates and improved yields.		
Reusability	MNCs can be reused multiple times without significant loss of activity, making them cost‐effective and sustainable for industrial applications.	Potential Leaching	There can be issues with the leaching of the catalytic species or degradation of the magnetic core, reducing the catalyst's lifespan and requiring additional purification steps.
Controlled Catalytic Properties	Surface modification of MNCs allows for tailored catalytic properties, enhancing selectivity and activity for specific reactions.		
Reduced Environmental Impact	The use of MNCs can lead to milder reaction conditions and lower energy consumption, aligning with green chemistry principles.	Cost and Resource Intensive	The preparation of highly specific and functionalized magnetic nanocatalysts can be resource‐intensive, increasing the overall cost of the catalytic process.
Versatility	MNCs can be applied in a wide range of reactions, including oxidation, reduction, and coupling reactions, making them versatile catalysts.		
Magnetic Field Assistance	The application of a magnetic field can enhance mass transfer rates, promoting the movement of reactants towards the catalyst surface.		

### 3.1 Challenges and Future Directions

Traditional synthesis methods in organic chemistry and catalysis have been vital for industrial and laboratory processes but often face efficiency, selectivity, and environmental sustainability challenges. Magnetic nanocatalysts have emerged as a promising alternative, addressing these issues by enhancing reaction efficiency and simplifying catalyst recovery through magnetic separation. Efforts towards sustainable chemistry have led to greener methods for C−S and C−Se bond formation, utilizing environmentally friendly solvent systems like water, ethanol, and deep eutectic solvents (DES). Despite progress, challenges persist in optimizing selectivity reactivity, developing nontoxic catalysts, and extending these techniques to a broader range of substrates with sensitive functional groups.[^1^, ^2^, ^3^, ^4^, ^5^, ^6^, ^7^, ^8^, ^9^, ^10^]

Conventional synthesis methods face significant challenges, primarily inefficiency in reaction yield and selectivity. These methods often require harsh conditions, such as high temperatures and pressures, and use stoichiometric amounts of reagents, leading to unwanted by‐products. This reduces process efficiency and raises environmental concerns due to waste generation and the use of toxic solvents. Additionally, traditional heterogeneous catalytic systems struggle with catalyst recovery and reuse, as separating catalysts from reaction mixtures can be complex and time‐consuming, increasing costs and environmental waste.[^15^, ^16^, ^17^, ^18^, ^19^, ^20^]

Magnetic nanocatalysts are utilized in various fields, including synthesizing fine chemicals, pharmaceuticals, polymers, and environmental cleanup processes like water purification and pollutant degradation. Their multifunctionality and ease of recovery make them promising candidates for sustainable catalytic processes across industries. However, challenges remain, such as the stability of nanocatalysts under reaction conditions, potential leaching of catalytic species, and the need for precise control over nanoparticle size and distribution. Ongoing research focuses on innovative catalyst design strategies, including developing more robust support materials and surface modifications to enhance the stability and functionality of magnetic nanocatalysts.[^21^, ^22^, ^23^, ^24^, ^25^, ^26^, ^27^, ^28^, ^29^, ^30^]

The application of magnetic nanocatalysts faces several critical challenges, particularly regarding their stability and reusability over multiple reaction cycles. Factors such as nanoparticle agglomeration, leaching of catalytic species, and chemical degradation can lead to deactivation. Innovative surface modifications and robust core materials are needed to enhance their longevity.[^31^, ^32^, ^33^, ^34^, ^35^]

Scaling up production for industrial applications also presents challenges, including maintaining consistency in particle size and catalytic properties and developing cost‐effective synthesis methods without compromising quality. While magnetic nanocatalysts align with green chemistry principles due to their reusability, their production may generate hazardous by‐products, and the long‐term environmental impact of released nanoparticles is still uncertain. Safe handling and disposal protocols are essential to mitigate health and environmental risks.[^50^, ^51^, ^52^, ^53^, ^54^, ^55^, ^56^, ^57^, ^58^, ^59^, ^60^]

Another limitation is their narrow substrate scope, which can restrict their effectiveness in various reactions. Factors such as catalyst incompatibility with specific functional groups and the need for extensive optimization complicate their use. Ongoing research aims to broaden their applicability through improved designs and tailored reaction conditions.[^70^, ^71^, ^72^, ^73^, ^74^, ^75^, ^76^, ^77^, ^78^, ^79^, ^80^] Emerging trends in the field include the development of hybrid nanocatalysts that combine magnetic materials with other functional nanomaterials to enhance performance and stability. Advanced synthetic techniques are also being explored for precise control of nanocatalyst properties.[^85^, ^86^, ^87^, ^88^, ^89^, ^90^] Interdisciplinary collaboration is crucial for advancing magnetic nanocatalysts, as it can lead to innovative synthesis methods and a better understanding of catalytic mechanisms. Partnerships between academia and industry are vital for translating research into commercial technologies.[^90^, ^91^, ^92^, ^93^, ^94^, ^95^]

Future goals for magnetic nanocatalysis include expanding substrate scope, enhancing sustainability, and developing enantioselective catalysts for pharmaceutical applications. Their integration into industrial processes offers opportunities to improve efficiency, reduce costs, and promote sustainability through easy recovery and reuse, ultimately streamlining production and minimizing environmental impact. Continued efforts are necessary to optimize these systems′ scalability and economic viability for industrial adoption.[^101^, ^102^, ^103^, ^104^]

### 3.2 Integration into Continuous Flow Systems

- Enhanced Mixing and Reaction Control: Continuous flow systems allow for precise control over reaction conditions (temperature, pressure, and reactant concentration), which increases efficiency and consistency. Magnetic nanocatalysts can be applied within these systems to facilitate rapid mixing and optimize reaction rates.
- Efficient Catalyst Recovery: Magnetic nanocatalysts can be quickly recovered from reaction mixtures using magnetic separation techniques, enabling quick and efficient recycling within continuous flow setups. This reduces downtime associated with traditional catalyst separation methods and enhances overall process efficiency.
- Scalability and Space Efficiency: Continuous flow systems streamline production processes and require less reaction time than batch processes. Magnetic nanocatalysts can facilitate this scalability, allowing for easy adaptation to increased production demands without significantly increasing system size.
- Reduced Waste Generation: The continuous nature of the flow system, combined with the reusability of magnetic nanocatalysts, contributes to lower waste generation and more sustainable manufacturing practices. This aligns well with green chemistry principles, promoting environmentally friendly processes.

### 3.3 Economic Barriers for Large‐Scale Production

- High Initial Investment: Setting up continuous flow systems equipped with magnetic nanocatalysts often requires a substantial upfront investment in technology and infrastructure. This includes specialized equipment for flow chemistry and magnetic separation, which may discourage companies from adopting these innovations.
- Production Costs and Scale‐Up Challenges: Producing magnetic nanocatalysts on a large scale can involve high costs related to materials, synthesis methods, and quality control. Ensuring consistent particle size and distribution across large batches is crucial for performance but can be challenging.
- Market Acceptance: The transition from traditional batch processes to continuous flow systems requires operational protocols, training, and sometimes staffing changes. Resistance to change and a lack of familiarity with new technologies can hinder widespread acceptance in established industries.
- Economic Viability of Research and Development: Continuous efforts are necessary to optimize the design and functionality of magnetic nanocatalysts. R&D requires funding and resources, and if the economic benefits of integrating these catalysts into existing systems are not demonstrated, investment may be limited.
- Regulatory Challenges: Introducing new materials, including magnetic nanocatalysts, may face regulatory scrutiny regarding safety, environmental impact, and efficacy. Navigating these regulations can add time and cost to the development and implementation phases, impacting overall economic feasibility.

Integrating magnetic nanocatalysts into continuous flow systems offers promising advantages for enhancing efficiency and sustainability in chemical manufacturing. However, overcoming economic barriers such as high initial investments, production costs, market acceptance issues, and regulatory challenges is crucial for realizing the potential of these advanced catalytic systems in large‐scale applications. Continued research, collaboration, and investment will be essential to address these obstacles and facilitate the industry‘s widespread adoption of magnetic nanocatalysts.[^105^, ^106^, ^107^, ^108^]

### 3.4 Synthesis of C−Se bond

**Scheme 1:**
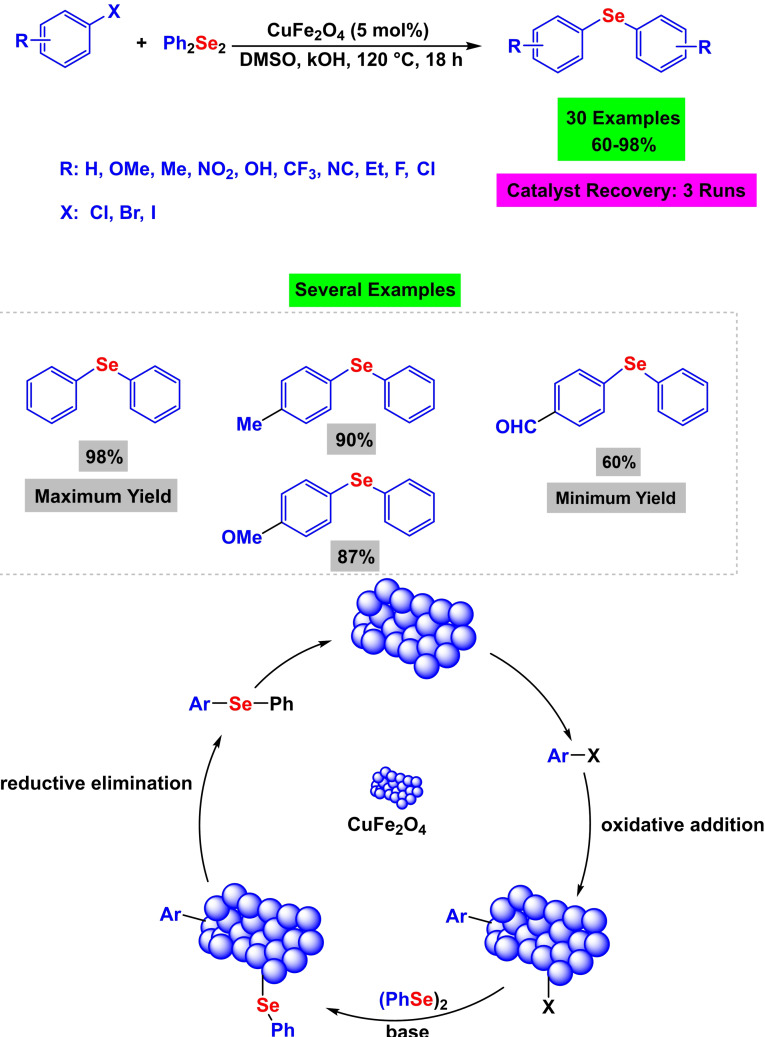
Synthesis of diphenylselane derivatives.

Using an external magnet, Yadavalli Venkata Durga Nageswar and his colleagues could effectively manufacture a magnetic and respirable CuFe_2_O_4_ nanoparticle in the year 2011 (Scheme 1). With the presence of 5 mol% CuFe_2_O_4_ in dimethyl sulfoxide anhydride as the solvent and potassium hydroxide as the base, the CuFe_2_O_4_ nanoparticle was put through its paces in C−Se coupling reactions in order to produce diaryl selenide. This was accomplished by reacting aryl halides with diphenyl selenide at 120 °C for 18 hours. Reusing the catalyst thrice was possible without seeing a significant decline in its activity.^[109]^

**Scheme 2:**
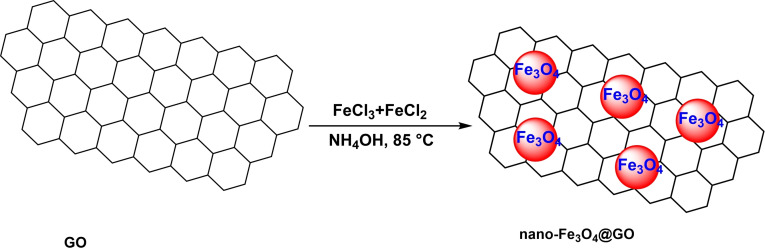
Synthesis f nano‐Fe_3_O_4_@GO nanoparticle.

**Scheme 3:**
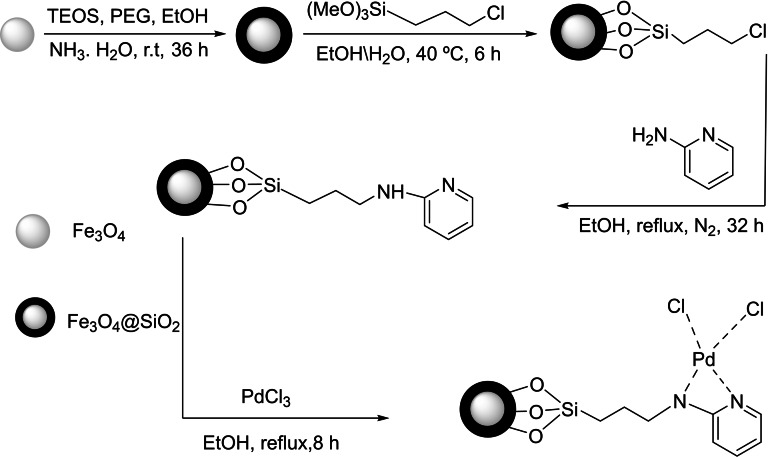
Synthesis of Fe_3_O_4_@SiO_2_/2‐aminopyridine‐Pd(II) as a magnetic nanocatalyst.

Immobilizing Fe_3_O_4_ on graphene oxide that had been synthesized using the co‐precipitation process as a magnetic nanocatalyst was a successful endeavor that Mohammad Zaman Kassaee and his colleagues accomplished in 2013 (Scheme 2). The nano‐Fe_3_O_4_@GO nanoparticle‘s structure was characterized using several analytical methods, including FT‐IR, X‐ray diffraction (XRD), transmission electron spectroscopy (TEM), and vibrating sample magnetometer (VSM). The VSM investigation revealed that the magnetic saturation value for Fe_3_O_4_ was 60 emu/g, whereas for nano‐ Fe_3_O_4_@GO, it was 21 emu/g. The reduction in the VSM curve may be related to the dispersion of Fe_3_O_4_ nanoparticles on the surface of GO and the fact that GO is an organic chemical that lacks saturation magnetism. The nano‐ Fe_3_O_4_@GO and Fe_3_O_4_ nanoparticles were used in C−Se coupling reactions to produce diaryl selenide (Scheme 3). This compound is created by the interaction of aryl halides with elemental selenium. The reaction occurred in the presence of 7 mol% nano‐ Fe_3_O_4_@GO as a catalyst, with dimethyl sulfoxide anhydride as the solvent and KOH as the base. The reaction was conducted at a temperature of 90 °C. The nano‐Fe_3_O_4_@GO exhibited superior performance to Fe_3_O_4_, mostly due to its high yields and low response times. The nano‐ Fe_3_O_4_@GO yielded 98 % over a 4‐hour timeframe, while Fe_3_O_4_ only yielded 63 % over 24 hours. The catalyst was used for four consecutive cycles with little reduction in its activity.^[110]^

In the year 2022, Xiaoqing Xu and his coworkers used Fe_3_O_4_@SiO_2_/2‐aminopyridine‐Pd(II) as a magnetic nanocatalyst. This was accomplished by first immobilizing a 2‐aminopyridine ligand on Fe_3_O_4_ that had been synthesized using the co‐precipitation technique at a temperature of 80 °C and then immobilizing palladium(II) on the ligand (Scheme 3).

**Scheme 4:**
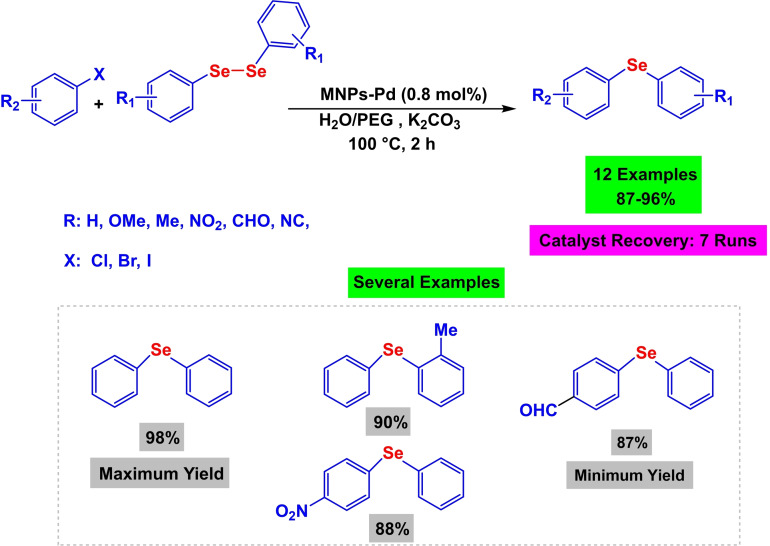
Synthesis of diaryl selenide scaffolds.

Using a variety of analytical methods, including infrared, transmission electron microscopy, scanning electron microscopy, thermogravimetric analysis, X‐ray diffraction, visual spectroscopy, and inductively coupled plasma optical emission spectroscopy, the structure of the nanoparticle‐based on palladium(II) was identified. In order to synthesize diaryl selenide, which was formed by the reaction of aryl halide, diselenide, and K_2_CO_3_ in H_2_O/PEG (1 : 1, 2 mL) at 100 degrees Celsius, the palladium(II)‐based nanoparticle was tested in C−Se coupling reactions (Scheme 4). The catalyst produced excellent yields, and it was separated by an external magnet and used seven times without experiencing a significant change in its activity.^[111]^

**Scheme 5:**
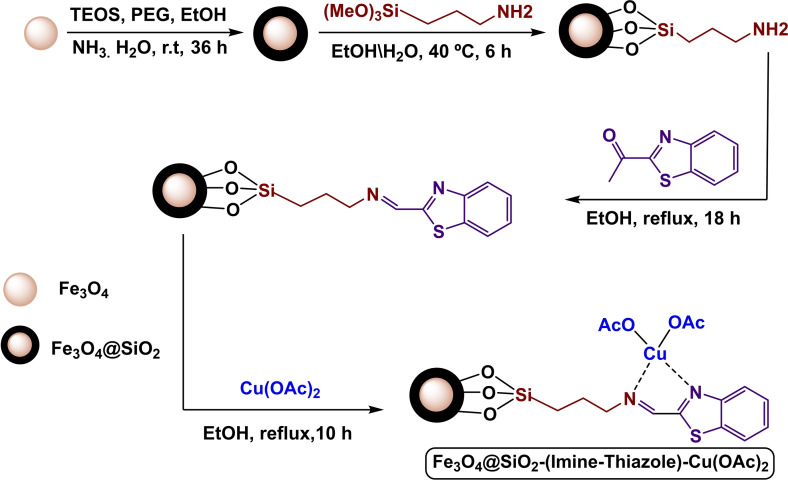
Synthesis of Fe_3_O_4_@SiO_2_‐(Imine‐Thiazole)‐Cu(OAc)_2_ nanocomposite.

**Scheme 6:**
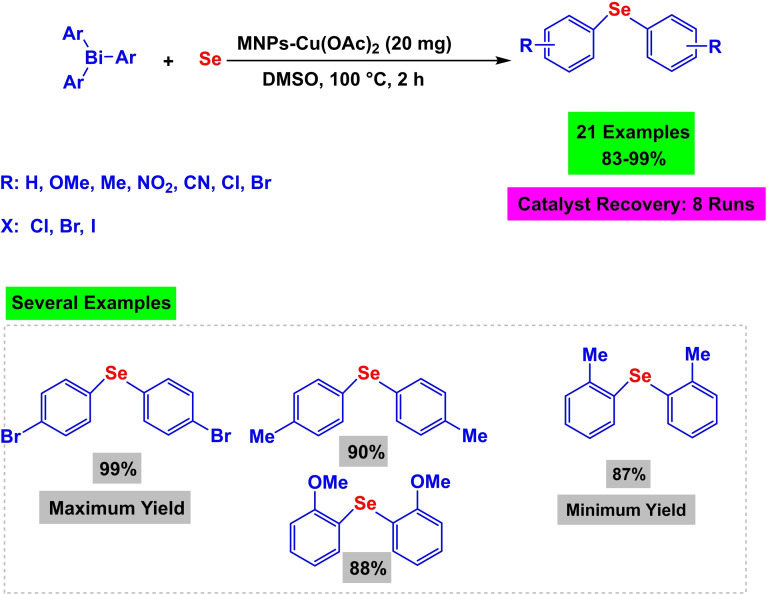
Synthesis of diaryl selenides.

**Scheme 7:**
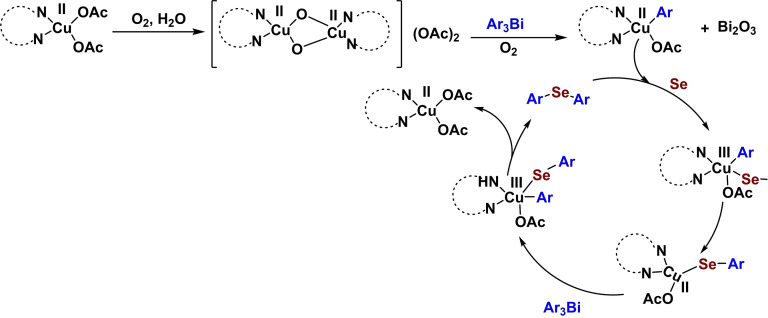
A plausible mechanism of synthesis of diaryl selenide scaffolds.

In 2023, Alshimaysawee Sadeq and co‐workers succeeded in synthesizing a magnetic and separable Fe3O4@SiO2‐(Imine‐Thiazole)‐Cu(OAc)_2_ nanocomposite by immobilizing Cu(OAc)_2_ on the Fe3O4nanocatalyst through Imine‐Thiazole as a ligand (Scheme 5). In order to determine the physical and chemical characteristics of the Fe_3_O_4_@SiO_2_‐(Imine‐Thiazole)‐Cu(OAc)_2_ nanocatalyst, spectroscopic procedures were carried out. These procedures included FT‐IR analysis, X‐ray diffraction (XRD), transmission electron spectroscopy (TEM), vibrating sample magnetometer (VSM), thermogravimetric analysis (TGA), and inductively coupled plasma mass spectrometry (ICP‐OES). The BET study discovered that the surface area is 5.89 cm^3^/g^−1^, and the pore size is 1.66 nm. The VSM analysis discovered that the magnetic saturation values of Fe_3_O_4_ and Fe3O4@SiO_2_‐(Imine‐Thiazole)‐Cu(OAc)_2_ are 65 and 50 emu/g, respectively. Both of these values were determined to be satisfactory. This decline occurred because of the effective encapsulation; nonetheless, it is still a modest decrease, indicating its relatively high magnetic value. C−Se coupling reactions were used to evaluate the Fe_3_O_4_@SiO_2_‐(Imine‐Thiazole)‐Cu(OAc)_2_ nanoparticle in order to manufacture diaryl selenide (Scheme 6). This was accomplished by combining Ph3Bi with Se powder in the presence of 20 mg of Fe_3_O_4_@SiO_2_‐(Imine‐Thiazole)‐Cu(OAc)_2_ in dimethyl sulfoxide anhydride as a solvent (Scheme 7). The reaction process took place at 100 °C for two hours. Reusing the catalyst eight times was possible without seeing a substantial decline in its function.^[112]^

**Scheme 8:**
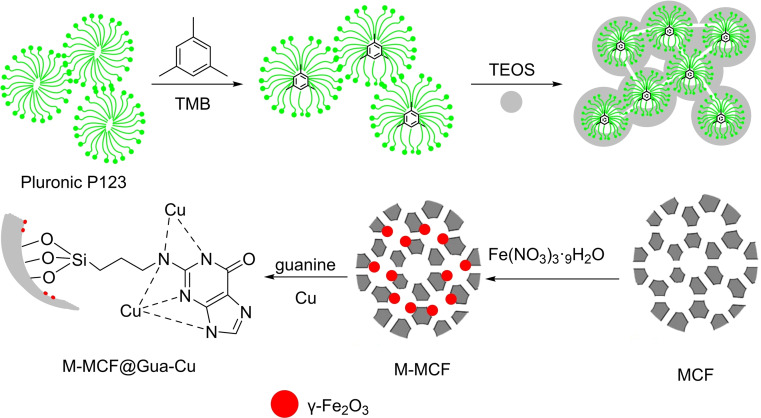
Synthesis of M–MCF@Gua‐Cu.

**Scheme 9:**
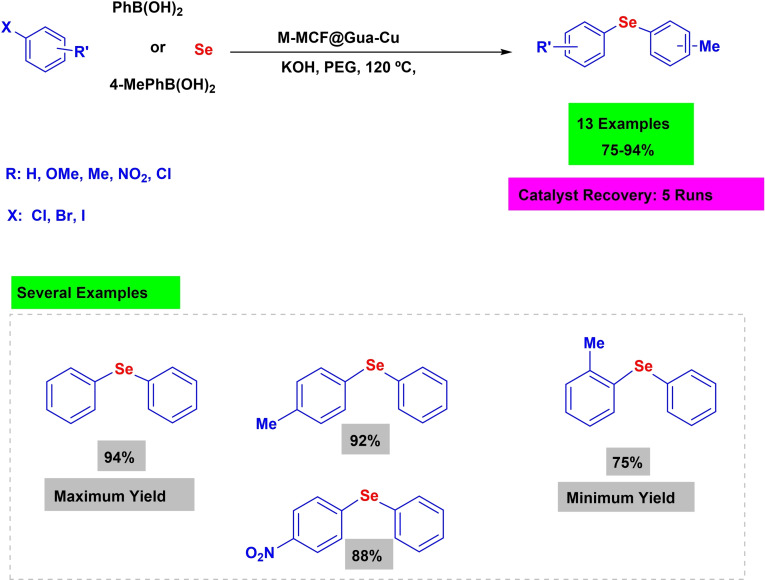
Synthesis of diaryl selenide derivatives.

**Scheme 10:**
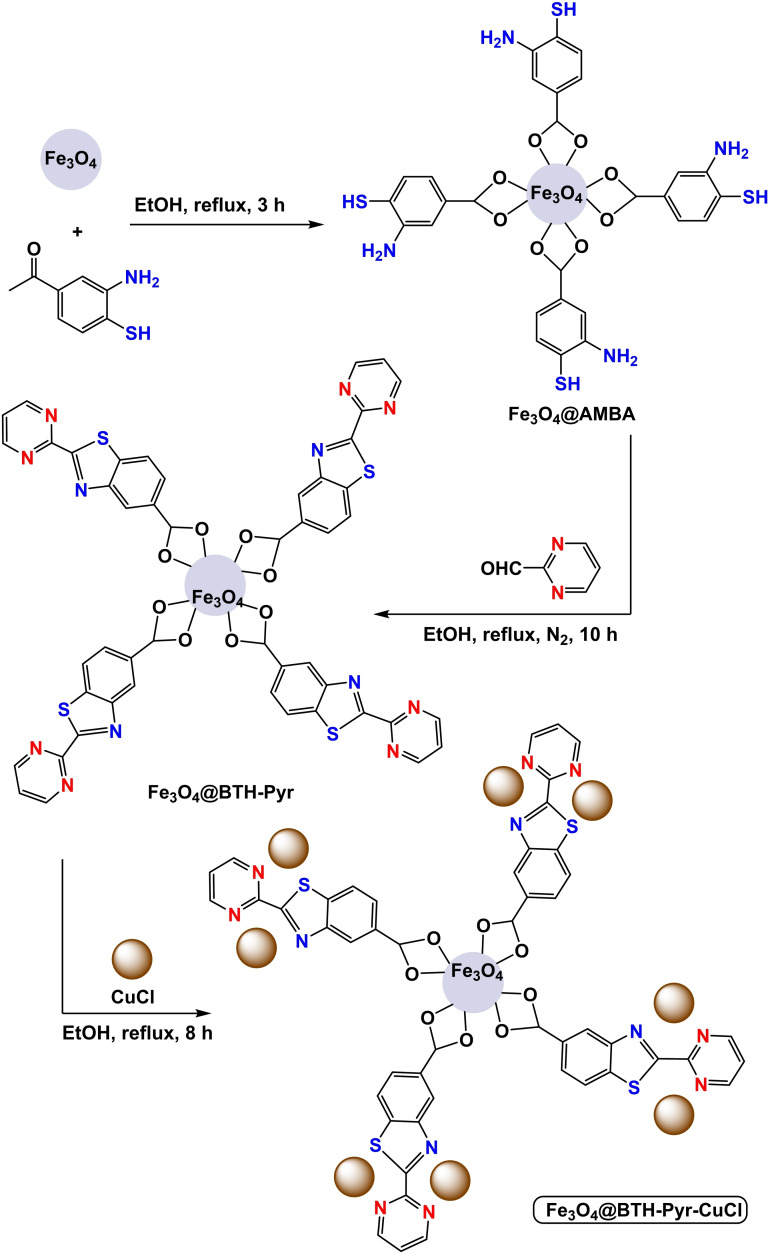
Synthesis of Fe_3_O_4_@BTH‐Pyr‐CuCl nanocatalyst.

**Scheme 11:**
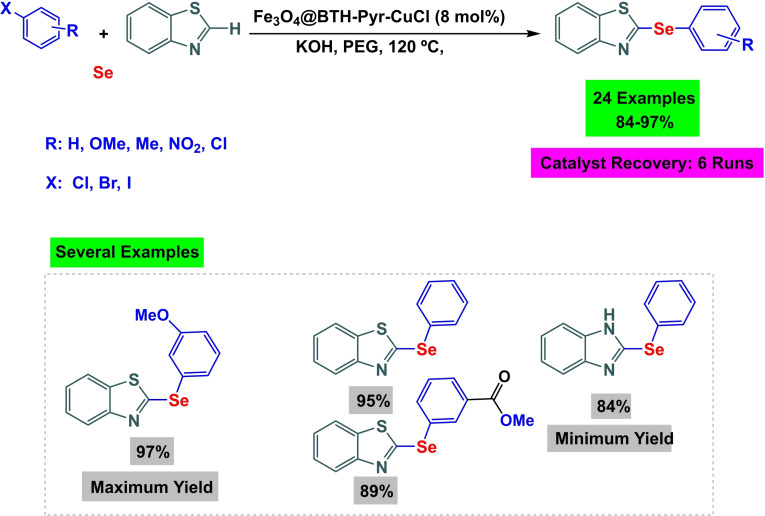
Synthesis of diaryl selenide compounds.

**Scheme 12:**
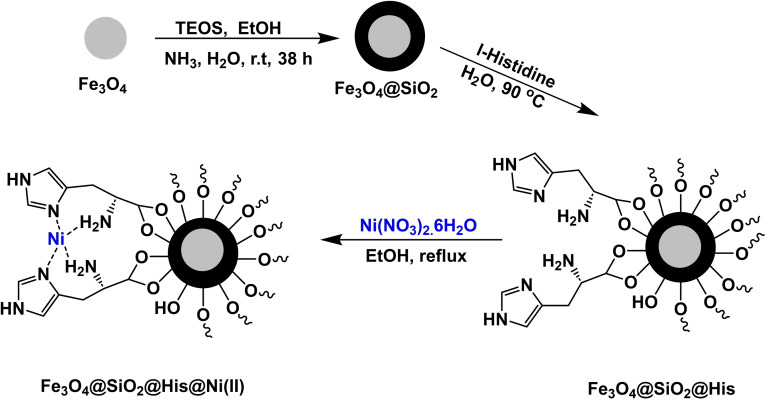
Synthesis of magnetic nanocatalyst Fe_3_O_4_@SiO_2_@His@Ni(II).

**Scheme 13:**
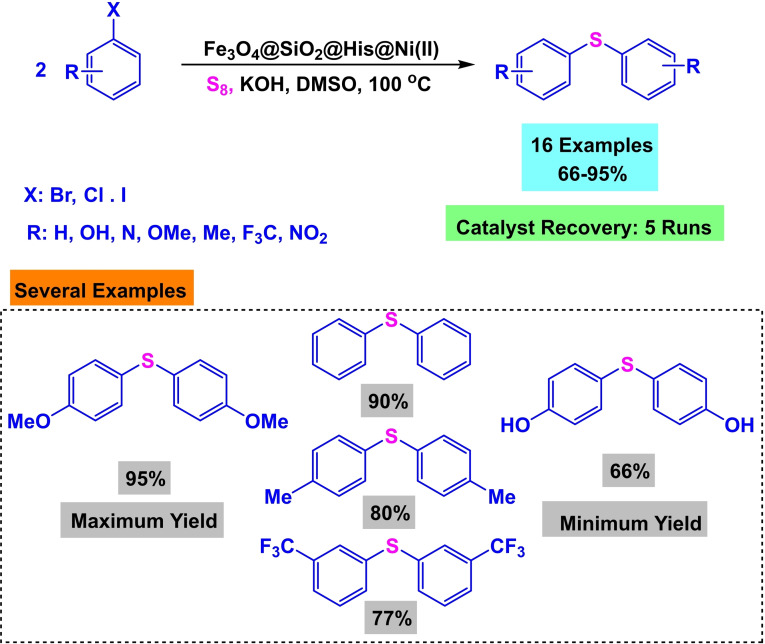
Synthesis of diphenyl sulfides.

**Scheme 14:**
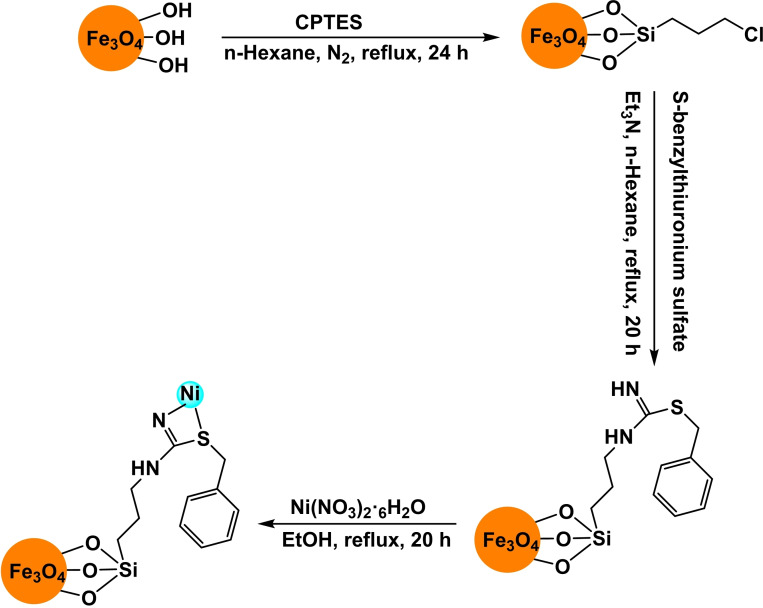
Synthesis of Fe_3_O_4_@SBTU@Ni(II).

**Scheme 15:**
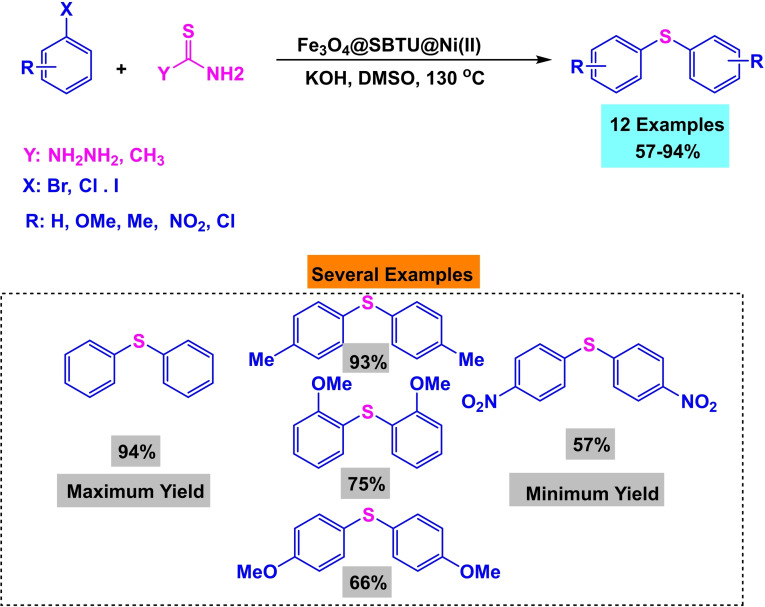
Synthesis of diphenyl sulfide scaffolds.

**Scheme 16:**
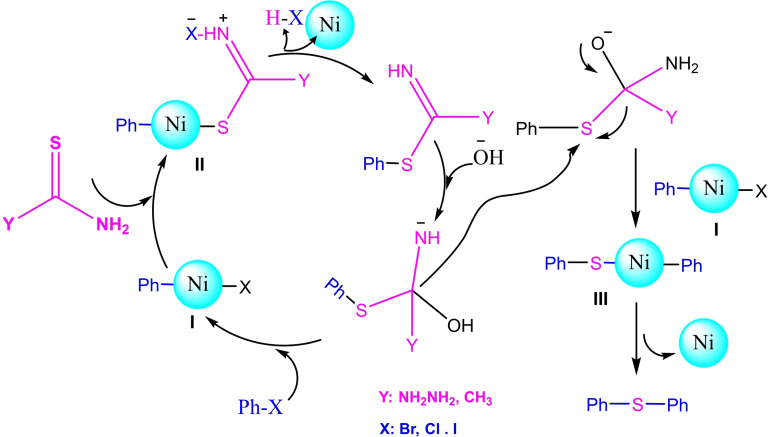
Mechanism of synthesis of diphenyl sulfide derivatives.

**Scheme 17:**
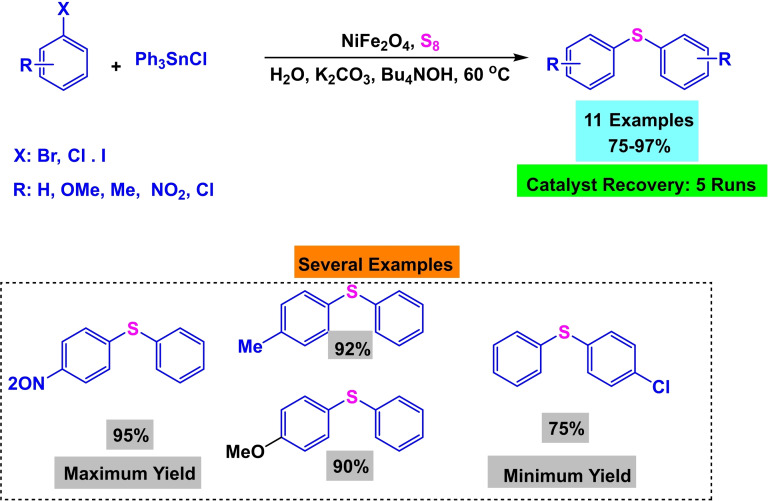
Synthesis of diphenyl sulfides.

**Scheme 18:**
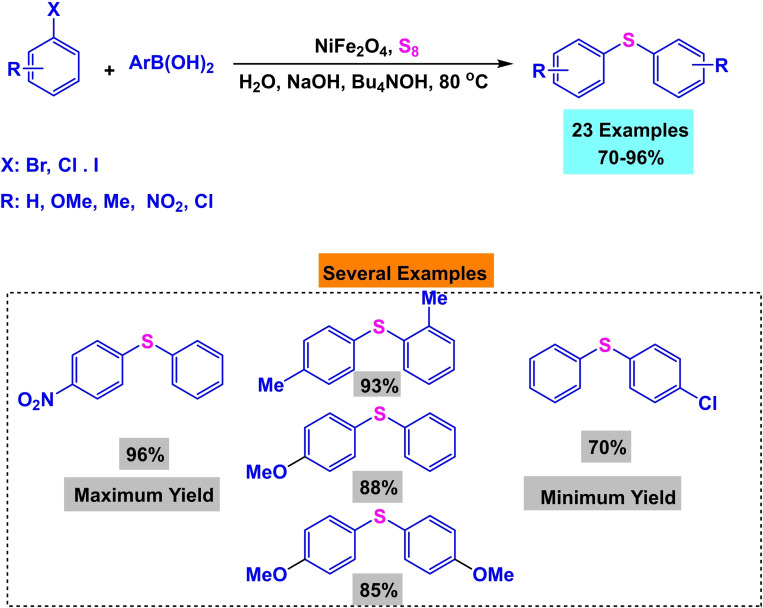
Synthesis of diphenyl sulfide moieties.

**Scheme 19:**
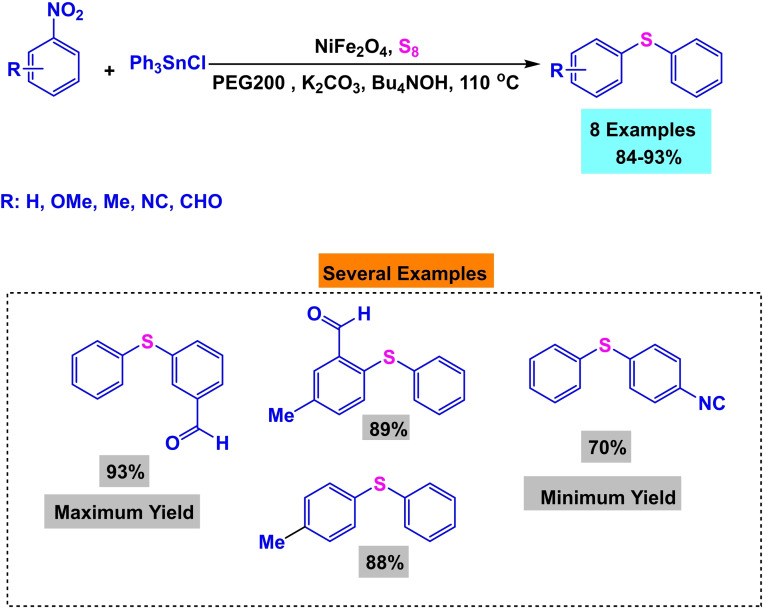
Synthesis of diphenyl sulfides.

**Scheme 20:**
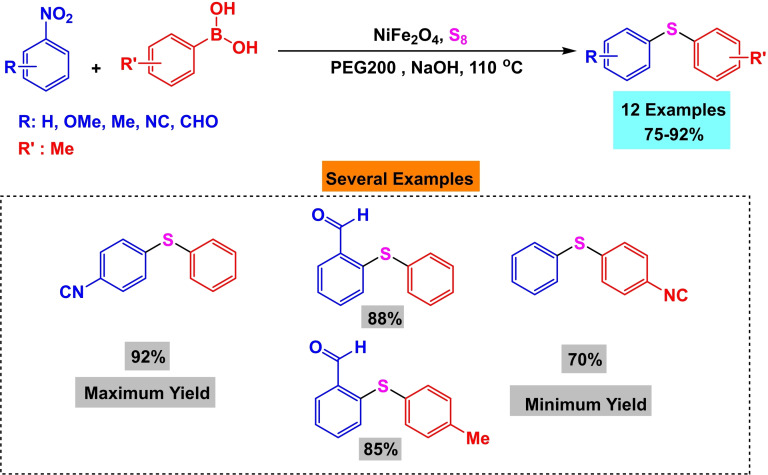
Synthesis of diphenyl sulfide compounds.

**Scheme 21:**
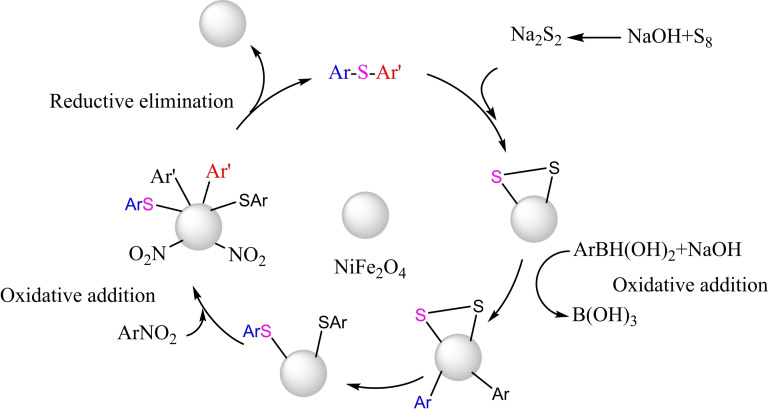
A plausible mechanism of synthesis of diphenyl sulfide deivatives.

**Scheme 22:**
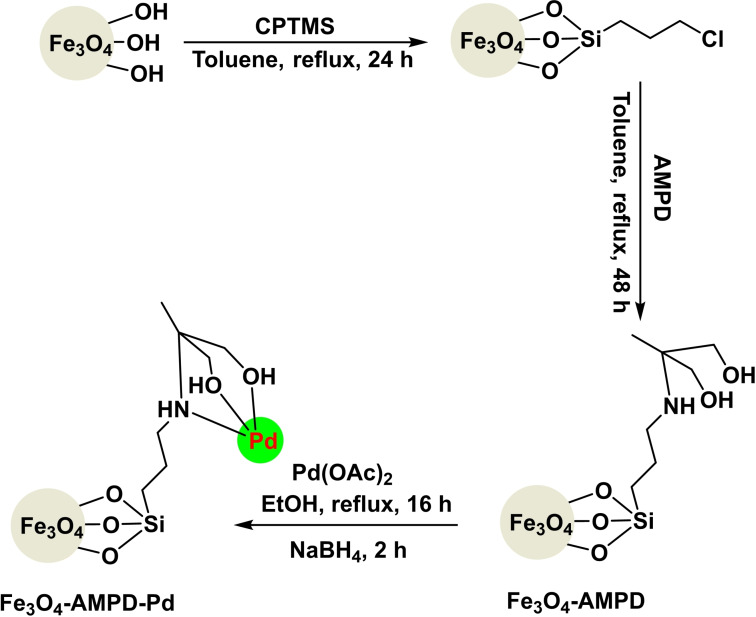
Synthesis of magnetic nanocatalyst Fe_3_O_4_‐AMPD−Pd.

**Scheme 23:**
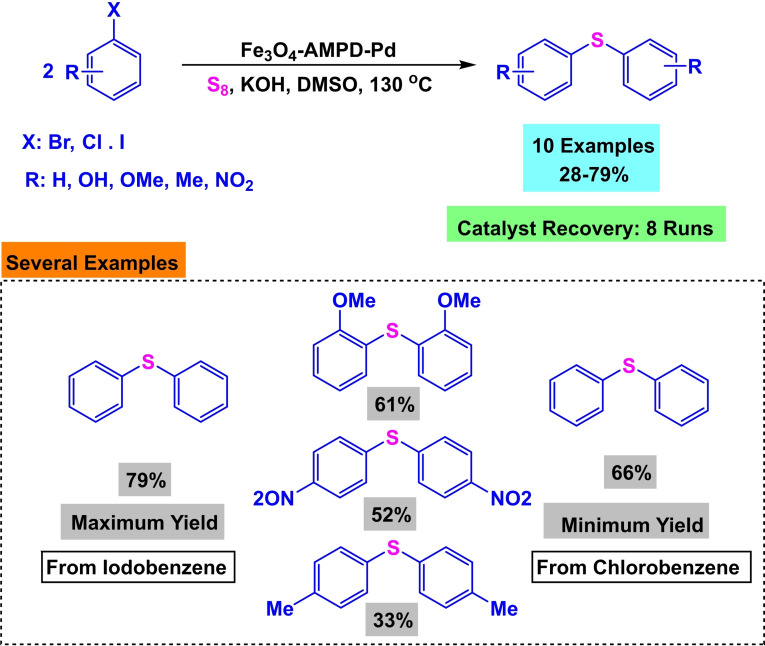
Synthesis of diphenyl sulfides.

**Scheme 24:**
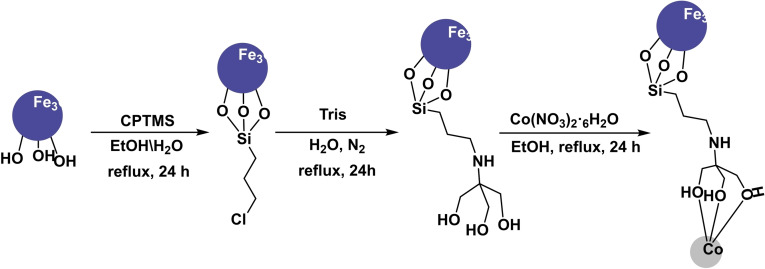
Synthesis of magnetic nanocatalyst Fe_3_O_4_@PTMS–Tris–Co.

In 2024, Amin Rostami and her coworkers developed a magnetic nanocatalyst called M–MCF@Gua‐Cu. The catalyst consists of γ‐Fe_2_O_3_ (referred to as M) and mesocellular silica (referred to as MCF). Initially, magnetic nanoparticles were created and inserted into the pores of MCF (Scheme 8). Subsequently, copper was fixed onto guanine. The structural characteristics of the copper‐based nanoparticle were examined using several analytical methods, including infrared spectroscopy (IR), high‐resolution transmission electron microscopy (HR‐TEM), scanning electron microscopy (SEM), thermogravimetric analysis (TGA), energy‐dispersive X‐ray spectroscopy (EDX), X‐ray diffraction (XRD), and vibrating sample magnetometry (VSM). The efficacy of a copper‐based nanoparticle, M–MCF@Gua‐Cu, was evaluated in C−Se coupling reactions. These reactions involved the synthesis of diaryl selenide through the reaction of aryl halides with either phenylboronic acid or 4‐methylphenylboronic acid, along with selenium powder. The reactions were conducted in the presence of 30 mg of M–MCF@Gua‐Cu as a catalyst, using PEG‐200 as the solvent and KOH as the base. The reactions were carried out at a temperature of 120 °C. Diaryl selenides were produced with high yields ranging from 70 % to 94 %. The catalyst was used for five cycles without substantially impacting its activity (Scheme 9, 10, 11, 12, 13, 14, 15, 16, 17, 18, 19, 20, 21, 22, 23, 24).^[113]^

In 2024, Ke Wang and co‐workers successfully created a magnetic and separable external magnet Fe_3_O_4_@BTH‐Pyr‐CuCl nanocomposite by attaching CuCl to the Fe_3_O_4_ nanocatalyst using a benzothiazole‐pyrimidine ligand. The Fe_3_O_4_@BTH‐Pyr‐CuCl nanocatalyst was analyzed using various spectroscopic techniques, including FT‐IR analysis, X‐ray diffraction (XRD), transmission electron spectroscopy (TEM), vibrating sample magnetometer (VSM), thermogravimetric analysis (TGA), and inductively coupled plasma mass spectrometry (ICP‐OES) to determine its physical and chemical properties. The ICP‐OES analysis revealed that the nanocatalyst structure contains 14.25×10–5 molg^−1^ of copper. The VSM study determined that the magnetic saturation value of Fe_3_O_4_ is 61.85 emu/g, whereas the magnetic saturation value of Fe_3_O_4_@BTH‐Pyr‐CuCl is 45.875 emu/g. The drop in value may be attributed to the effective encapsulation, albeit relatively small, indicating its strong magnetic properties. The Fe_3_O_4_@BTH‐Pyr‐CuCl nanoparticle was evaluated in C−Se coupling reactions to synthesize diaryl selenide. This involved the reaction of aryl iodides with Se powder or heteroaryl in the presence of 20 mg of Fe_3_O_4_@BTH‐Pyr‐CuCl. The reaction was conducted at 120 °C for 4 hours using KOAc as a base and PEG‐400 as a solvent. The catalyst was used for six cycles without any noticeable decline in its activity.^[114]^

### 3.5 Synthesis of S‐C bond

Ghorbani‐Choghamarani and his research team successfully constructed a magnetic nanocatalyst Fe_3_O_4_@SiO_2_@His@Ni(II) in 2017. The magnetic core was generated using co‐precipitation and then coated with SiO_2_. In order to stabilize and trap Ni(II), l‐histidine was added as a ligand to stabilize Ni(II). FT‐IR, SEM, XRD, and TGA identified the magnetic nanocatalyst Fe_3_O_4_@SiO_2_@His@Ni(II). It was utilized in the production of diphenyl sulfides to evaluate the effectiveness of the catalyst Fe_3_O_4_@SiO_2_@His@Ni(II). This was accomplished by reacting aryl halide with S8 in the presence of DMSO as a solvent and KOH as a base while using 0.04 grams of catalyst at 100 degrees Celsius. During five successive cycles, the activity of the catalyst was evaluated, and it was found that it did not lose a substantial portion of its activity.^[115]^

In a research paper published the same year, Arash Ghorbani‐Choghamarani and his team created Fe_3_O_4_ magnetic nanoparticles supported by 3‐Chloropropyltriethoxysilane (CPTES). They then added S‐benzylthiuronium sulfate as a ligand to stabilize Ni(II) by introducing Ni(NO_3_)_2_⋅6H_2_O to the Fe_3_O_4_@SBTU mixture. This resulted in forming a new nanocatalyst called Fe_3_O_4_@SBTU@Ni(II). The catalyst underwent characterization utilizing various analytical techniques, including Fourier Transform Infrared Spectroscopy (FT‐IR), Vibrating Sample Magnetometry (VSM), Scanning Electron Microscopy (SEM), X‐ray Diffraction (XRD), and Thermogravimetric Analysis (TGA). The XRD examination reveals that the magnetic core possesses a high degree of crystallinity and retains its crystalline structure as a cubic spinel, even after adding amorphous organic components. The VSM study reveals that the catalyst Fe_3_O_4_@SBTU@Ni(II) exhibits a significant level of magnetism, with a saturation value of 30 emu/g. This strong magnetism enables the catalyst to be easily separated using an external magnet. The catalyst‘s efficacy was evaluated in producing diaryl/alkyl sulfides by the aryl/alkyl halide, thioacetamide, and thiosemicarbazide reactions. This reaction occurred at 130 °C, with KOH as the base and DMSOF as the solvent. The catalyst exhibited significant production rates of diaryl sulfides, ranging from moderate to high.^[116]^

In 2017, Amin Rostami and his research team published a paper detailing the synthesis of a magnetic nanocatalyst nickel ferrite (NiFe_2_O_4_). The co‐precipitation process created the nanocatalyst from the reaction of iron and nickel chlorides. Multiple reactions demonstrated the efficacy of the catalyst. First, Phenylaryl sulfides were synthesized by reacting triphenyltin chloride, S_8_, and aryl halides in the presence of Bu_4_NOH, K_2_CO_3_, and water as a solvent at 80 °C for 15 to 32 hours. The yields obtained ranged from 75 % to 97 %. Then, diaryl halides were synthesized by reacting aryl halides, aryl boronic acids, and S8 in the presence of Bu_4_NOH and NaOH in water at a temperature of 80 °C for a duration of 13 to 34 hours. The resulting yields ranged from 70 % to 95 %. Then, Phenylaryl sulfides were synthesized by reacting triphenyltin chloride, nitroarene, and S8 in the presence of potassium carbonate and PEG200 as a solvent at a temperature of 110 °C for 8 to 20 hours. The yields obtained ranged from 84 % to 93 %. Then, the unsymmetric diaryl sulfides were synthesized by reacting nitroarene, aryl boronic acid, and S_8_ in the presence of NaOH and PEG200 as a solvent at a temperature of 110 °C for 11 to 34 hours. The yields obtained ranged from 75 % to 92 %. The catalyst was successfully utilized five times in synthesizing 4‐nitro anisole using triphenyltin chloride without any noticeable decline in its catalytic efficiency.^[117]^

In 2018, Arash Ghorbani‐Choghamarani and his team successfully developed a magnetic nanocatalyst called Fe_3_O_4_‐AMPD−Pd. The catalyst consisted of Fe_3_O_4_ as the magnetic core and basic substrate. CPTMS was introduced, adding of 2‐amino‐2‐methyl‐1,3‐propanediol (AMPD) as a stabilizing ligand for Pd. The catalyst was evaluated using various techniques, including X‐ray diffraction (XRD), transmission electron microscopy (TEM), thermogravimetric analysis (TGA), Fourier‐transform infrared spectroscopy (FT‐IR), and vibrating sample magnetometry (VSM). The catalyst‘s efficiency was demonstrated by synthesizing diphenyl sulfides by the reaction of aryl halide and S_8_ as a sulfur source in the presence of KOH as a base, DMSO as a solvent, and 0.06 g of Fe_3_O_4_‐AMPD−Pd as a catalyst. The reaction was carried out at 100 °C for 40 to 310 minutes, resulting in yields ranging from 28 % to 79 %. The catalyst was employed for eight cycles in synthesizing of diphenyl sulfides without any noticeable decline in catalytic efficiency.^[118]^

During the year 2019, Wan‐Xi Peng and his team developed a magnetic nanocatalyst Fe_3_O_4_@PTMS–Tris–Co. This nanocatalyst utilized Fe_3_O_4_ as a magnetic core and was generated using co‐precipitation. CPTMS was added, and Tris was finally grafted to stabilize the Co. The catalyst that was synthesized underwent characterization using various techniques, including X‐ray diffraction (XRD), transmission electron microscopy (TEM), thermogravimetric analysis (TGA), Fourier‐transform infrared spectroscopy (FT‐IR), and vibrating sample magnetometry (VSM). The catalyst‘s efficiency was demonstrated by performing S‐arylation preparation using aryl halide and thiourea reaction in the presence of K_2_CO_3_ as a base, EtOH as a solvent, and 0.40 mol% Fe_3_O_4_@PTMS‐Tris‐Co as a catalyst under reflux conditions for 5 to 60 minutes, resulting in yields ranging from 89 % to 100 %. The catalyst was successfully reused five times in the synthesis of diaryl sulfides without any noticeable decrease in catalytic activity.^[119]^

**Scheme 25:**
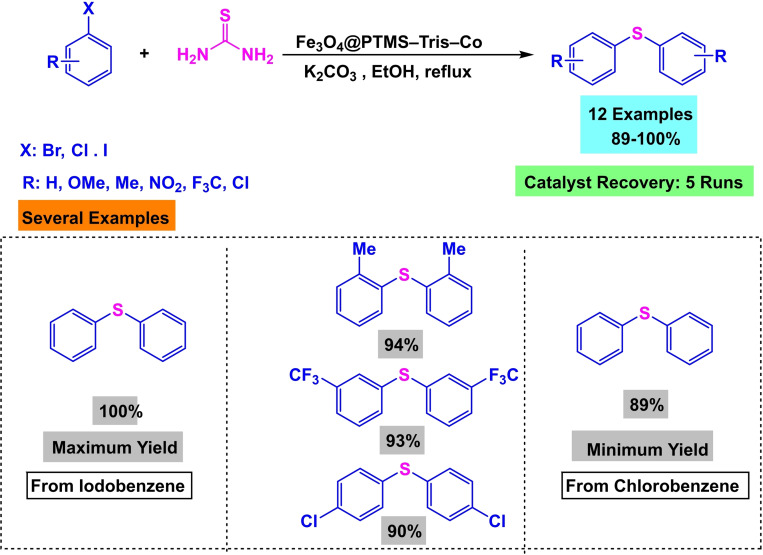
Synthesis of diphenyl sulfide scaffolds.

**Scheme 26:**
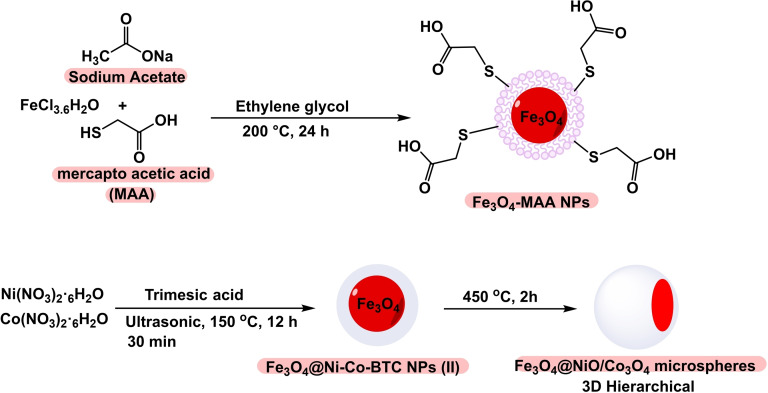
Synthesis of Fe_3_O_4_@NiO/Co_3_O_4_ microspheres.

**Scheme 27:**
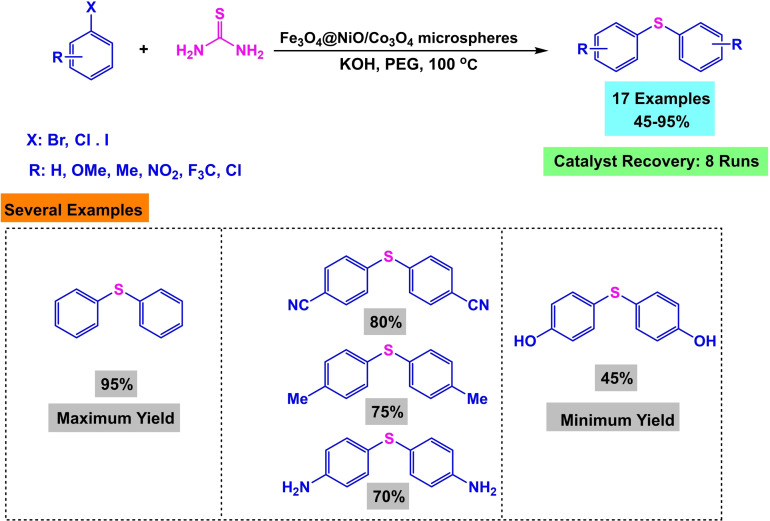
Synthesis of diaryl sulfides.

**Scheme 28:**
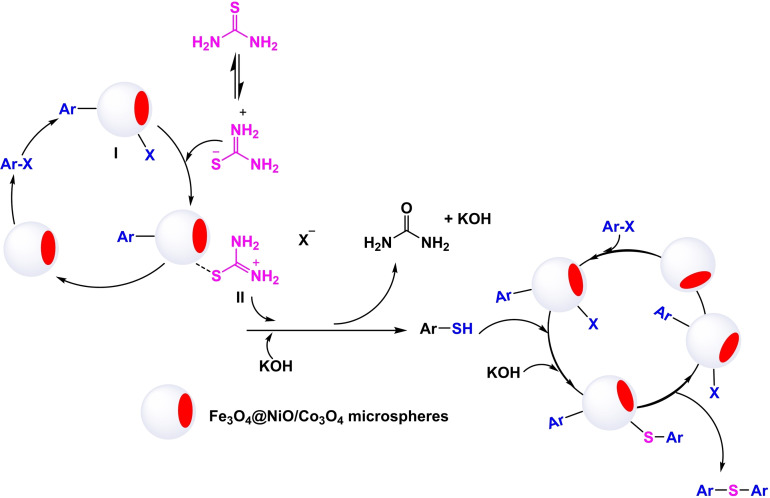
Mechanism of synthesis of diphenyl sulfides.

**Scheme 29:**
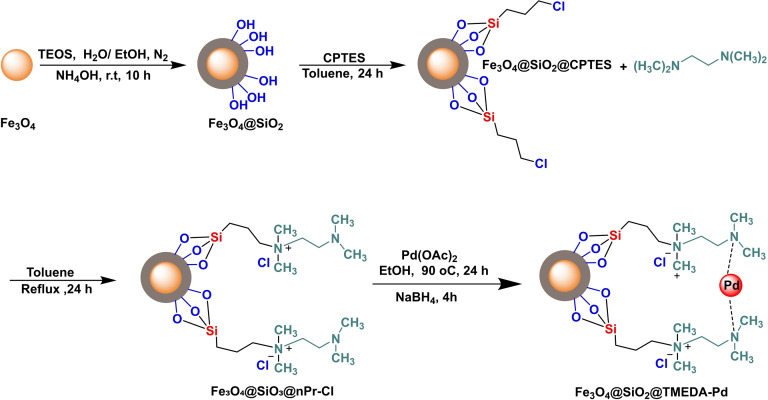
Synthesis of magnetic nanocatalyst Fe_3_O_4_@SiO_2_@TMEDA−Pd.

**Scheme 30:**
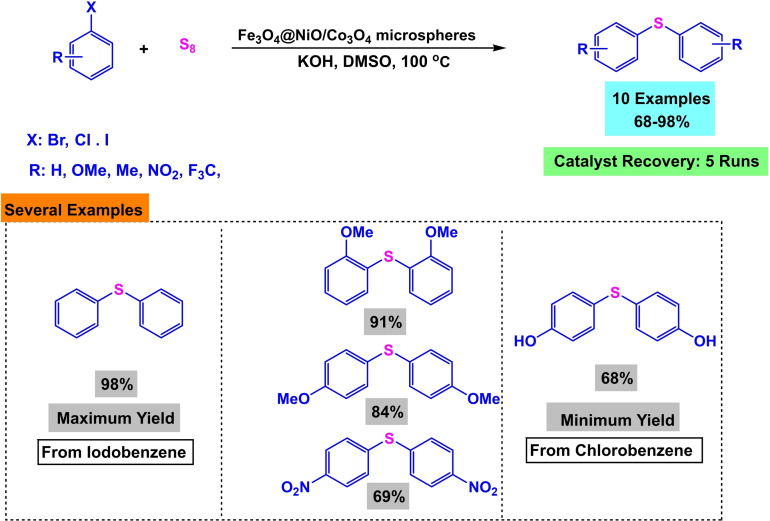
Synthesis of diphenyl sulfides.

In 2020, Batool Akhlaghinia and her team developed a magnetic nanocatalyst of 3D microspheres with a pyramidal core of Fe_3_O_4_@NiO/Co_3_O_4_ microspheres (Scheme 25). This nanocatalyst utilized Fe3O4 as a magnetic core, with the synthesis involving the initial formation of Fe_3_O_4_‐MAA nanoparticles. Mercaptoacetic acid (MAA) was employed to modify the surface and stabilize additional compounds. Subsequently, Fe_3_O_4_@Ni−Co‐BTC nanoparticles were synthesized via co‐precipitation, with thermic acid used to stabilize nickel and cobalt. The compound was then transformed into 3D microspheres by heating in air at 450 °C for 2 hours. The synthesized catalyst was characterized using Fourier transform infrared spectroscopy (FT‐IR). The catalytic efficiency was demonstrated through the preparation of diaryl sulfides via the reaction of aryl halides with thiourea in the presence of potassium hydroxide (KOH) as the base, polyethylene glycol (PEG) as the solvent, and Fe_3_O_4_@NiO/Co_3_O_4_ microspheres as the catalyst at 100 °C for durations ranging from 15 minutes to 24 hours, resulting in yields between 45 % and 95 %. The catalyst was successfully reused eight times in synthesizing diaryl sulfides without significantly declining catalytic activity (Scheme 26, 27, 28, 29, 30).^[120]^

In 2023, Alireza Kohzadian and his colleagues successfully synthesized a magnetic nanocatalyst compound, Fe_3_O_4_@SiO_2_@TMEDA−Pd. A co‐precipitation technique produced iron oxide (Fe_3_O_4_) nanoparticles and then modified by coating the surface with Fe_3_O_4_@SiO_2_. Furthermore, the particles underwent modification using 3‐chloropropyltrimethoxysilane (CPTMS), enhancing their surface characteristics. The third step was the attachment of N−N‐N′‐N′‐tetramethylethane‐1,2‐diamine (TMEDA) to the surface of the modified nanoparticles (Fe₃O₄@SiO_2_@nPr−Cl MNPs), thereby introducing amino functionalities that would augment the following reactions. The immobilization of palladium on Fe₃O₄@SiO₂@TMEDA nanoparticles has facilitated the use of these compounds as catalysts in various chemical processes. The catalyst was characterized using several techniques such as X‐ray diffraction (XRD), transmission electron microscopy (TEM), thermogravimetric analysis (TGA), Fourier transform infrared spectroscopy (FT‐IR), vibrating sample magnetism (VSM), inductively coupled plasma optical emission spectroscopy (ICP‐OES), and boundary element theory (BET). In order to evaluate the effectiveness of the catalyst, the arylation of S was carried out by reacting aryl halide and S_8_ in the presence of KOH as the base, DMSO and H_2_O as the solvent, and 0.89 mol% Fe_3_O_4_@PTMS‐Tris‐Co as the catalyst at 100 °C for 1.5 h to 10 h. The obtained yields varied between 68 % and 98 %. With no notable reduction in catalytic activity, the catalyst was effectively reused five times in producing diaryl sulfides.^[121]^

**Scheme 31:**
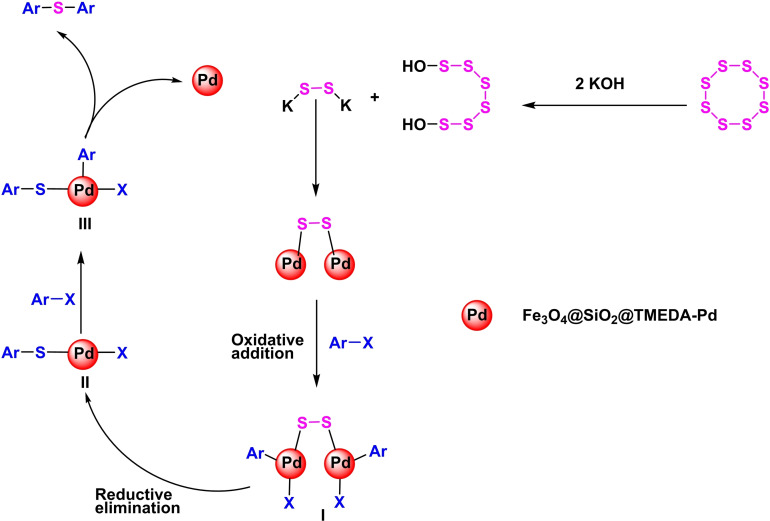
A plausible of mechanism of synthesis of diphenyl sulfides.

**Scheme 32:**
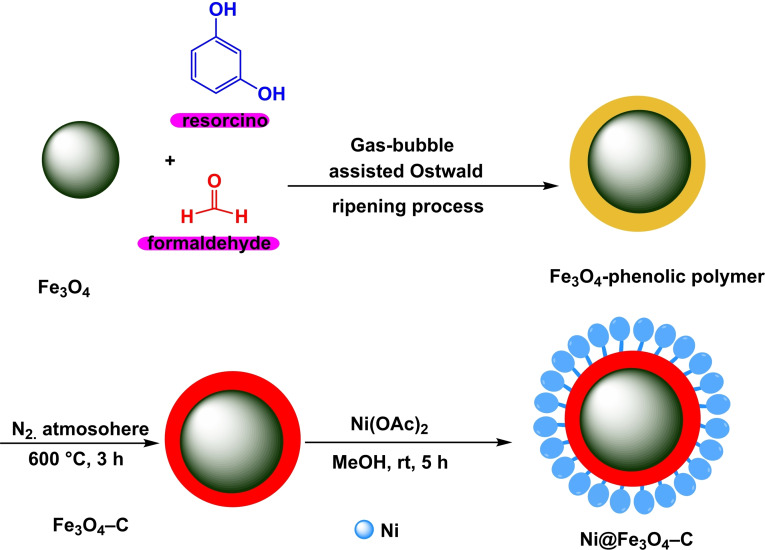
Synthesis of magnetic nanocatalyst called Ni@Fe_3_O_4_–C.

**Scheme 33:**
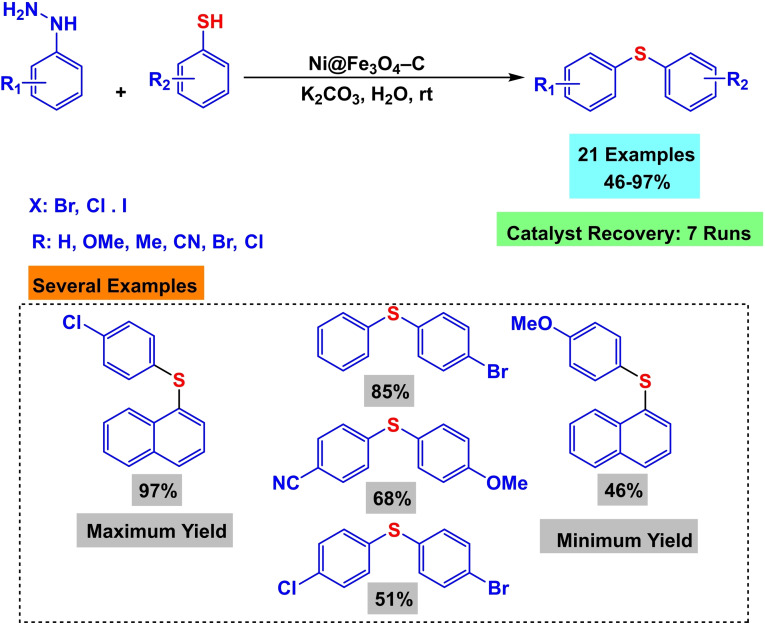
production of diallyl sulfides.

In 2024, Rakesh Kumar Sharma and his colleagues demonstrated the effective development of a magnetic nanocatalyst called Ni@Fe_3_O_4_–C. The production included a single‐step procedure in which solid Fe_3_O_4_ was melted and then converted into a circular magnetic nanoreactor with two hollow shells. The double‐shell hollow phenolic polymer was formed by the reaction of liquid ammonia as a structural agent and resorcinol‐formaldehyde as the carbon precursor. Multiple analytical techniques were employed to characterize the catalyst, including X‐ray diffraction (XRD), transmission electron microscopy (TEM), thermogravimetric analysis (TGA), Fourier transform infrared spectroscopy (FT‐IR), vibrating sample magnetometry (VSM), and X‐ray photoelectron spectroscopy (XPS). In order to evaluate the effectiveness of the catalyst, the researchers examined its performance in the production of diallyl sulfides (Scheme 31). An experimental reaction was conducted utilizing 4‐cyanophenylhydrazine hydrochloride and 4‐bromothiophenol as the substrate and K_2_CO_3_ as the solvent, with 25 mg Ni@Fe_3_O_4_–C acting as the catalyst. The reactions were conducted at ambient temperature for 20 hours, resulting in outcomes ranging from 46 % to 97 %. Significantly, the catalyst exhibited exceptional reusability, retaining constant catalytic activity during seven cycles in synthesizing diallyl sulfides (Scheme 32 and 33).^[122]^

In 2024, Maryam Sadat Ghorayshi and her colleagues successfully produced a magnetic nanocatalyst of Fe_3_O_4_ @xSiO_2_@ySiO_2_@BisPyP−Ni molecules. The manufacturing process included many sequential stages. The first stage included the production of the solid Fe_3_O_4_ core. During the second phase, the coating procedure began with applying tetraethylorthosilicate (TEOS) on two occasions. The first layer was applied to the nanoparticle Fe_3_O_4_ @ xSiO_2_, whereas the second layer was applied following the addition of cetyltrimethylammonium bromide (CTAB) Fe_3_O_4_ @ xSiO_2_@ySiO_2_. Finally, the combination was transformed into the) Fe_3_O_4_@ xSiO_2_@ySiO_2_@ nPr−Cl by adding 3‐Chloropropyltrimethoxysilane (CPTMS). In order to enhance the stability of nickel, the ligand 1,3‐Bis(4‐pyridyl) propane was used as the lead catalyst Fe_3_O_4_@ xSiO_2_@ySiO_2_@ BisPyP−Ni. Analytical methods such as X‐ray diffraction (XRD), transmission electron microscopy (TEM), thermogravimetric analysis (TGA), Fourier transform infrared spectroscopy (FT‐IR), vibrating sample magnetism (VSM), inductively coupled plasma (ICP), adsorption/desorption porosimetry (Brunauer‐Emmett‐Teller (BET)), and energy dispersive X‐ray spectroscopy (EDX) were employed to characterize the catalyst. X‐ray diffraction (XRD) and energy‐dispersive X‐ray spectroscopy (EDX) examination verified the sample‘s pristine crystallinity and the existence of nickel on the catalyst surface. However, in voltammetry (VSM) investigation, a slightly reduced magnetic saturation value was detected due to the development of organic compounds on the catalyst surface. The researchers assessed the efficacy of the catalyst via an investigation of its performance in synthesizing homodimeric diallyl sulfides. The reaction used aryl halides as sources, S_8_ and K_2_CO_3_ as bases, and PEG‐400 as the solvent. The catalyst used was 5 mg of Fe_3_O_4_@ xSiO_2_@ySiO_2_@ BisPyP−Ni. The reactions were conducted at a temperature of 80 °C for 20–140 minutes, resulting in yields ranging from 79 % to 98 %.^[123]^

**Scheme 34:**
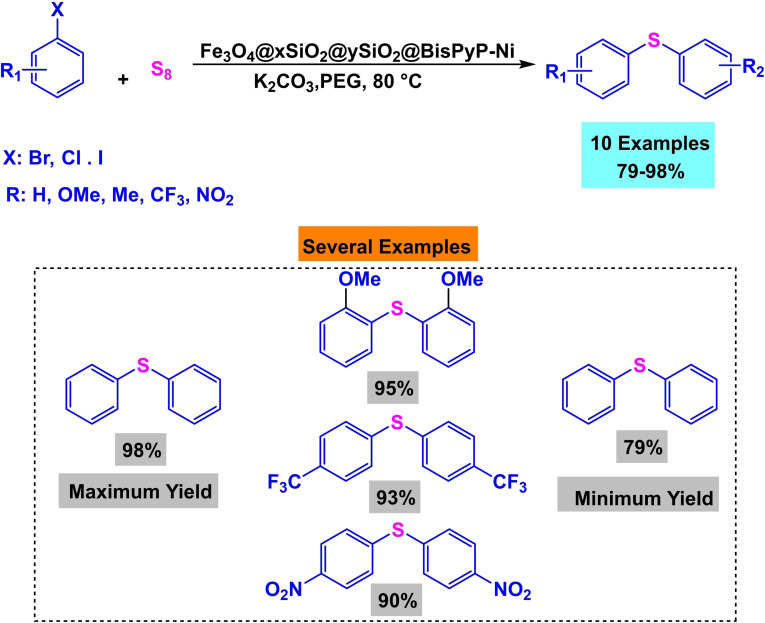
Synthesis of diphenyl sulfides.

**Scheme 35:**
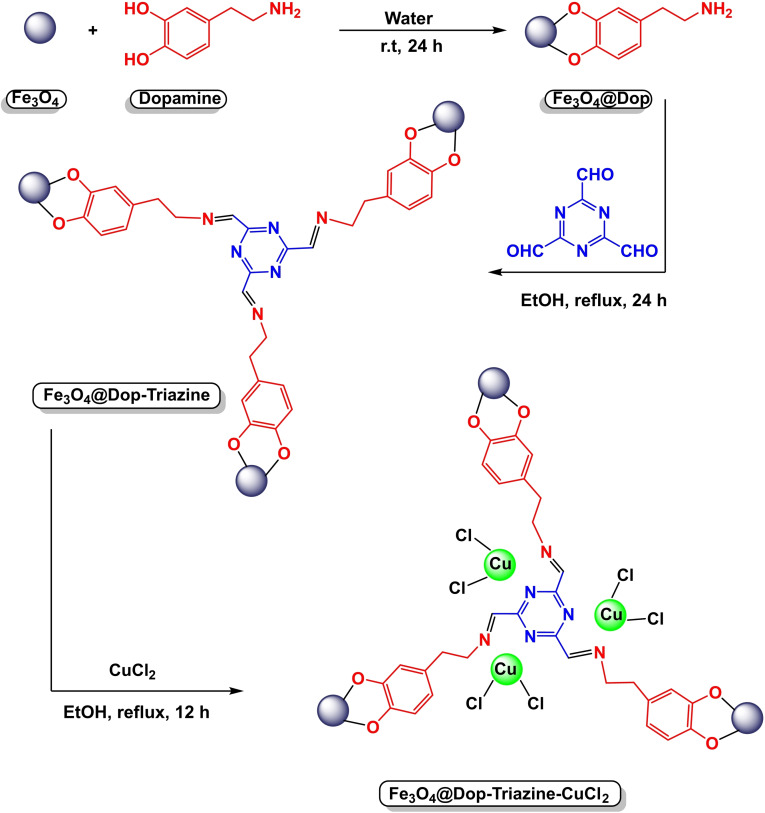
Preparation of magnetic nanocatalyst Fe3O4@Dop‐Triazine‐CuCl_2_.

**Scheme 36:**
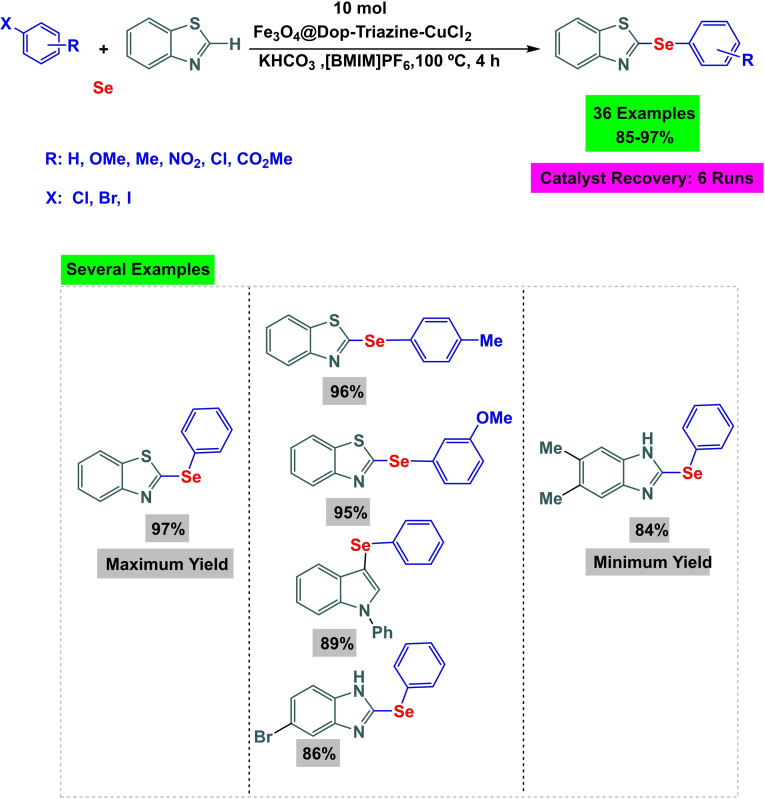
Synthesis of diphenyl sulfides.

**Scheme 37:**
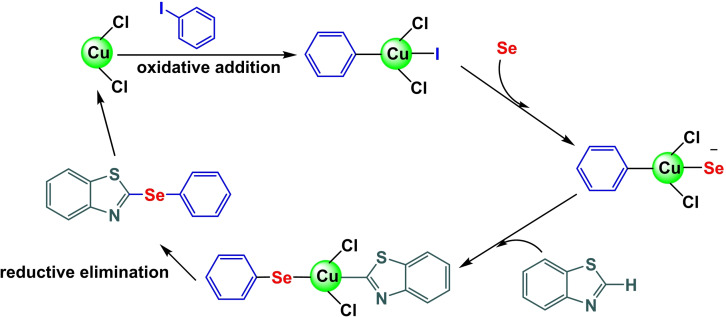
Mechanism of synthesis of diphenyl sulfide compounds.

In 2024, Yuxuan Lu and co‐workers developed an efficient magnetic nanocatalyst Fe_3_O_4_@Dop‐Triazine‐CuCl_2_. The procedure firstly involved preparing magnetic nanoparticles of Fe_3_O4 and coating them with dopamine to make Fe_3_O_4_@Dop nanocomposite (Scheme 34). Then, due to the magnetic interaction of Fe_3_O_4_@Dop nanocomposite with 1,3,5‐Triazine‐2,4,6‐tricarbaldehyde, imine bonds are formed and the magnetic nanocomposite [Fe_3_O4@Dop‐Triazine] is well constructed. Finally, by immobilizing copper(II) chloride on the Fe_3_O_4_@Dop‐Triazine ligand, the target nanocatalyst [Fe3O4@Dop‐Triazine‐CuCl_2_] was successfully fabricated. Multiple analytical techniques were used to characterize the catalyst, including X‐ray diffraction (XRD), transmission electron microscopy (TEM), thermogravimetric analysis (TGA), Fourier transform infrared spectroscopy (FT‐IR), vibrating sample magnetism (VSM), and X‐ray photon spectroscopy (XPS). In order to evaluate the effectiveness of the catalyst, the researchers examined its performance in producing aryl(heteroaryl)‐heteroaryl selenides through C−Se coupling reactions (Scheme 34). A pilot reaction was carried out using benzothiazole, iodobenzene, selenium powder, and KHCO_3_ as the base, and [BMIM]PF6 as the solvent with 10 mol% Fe_3_O_4_@Dop‐Triazine‐CuCl_2_ as the catalyst at 100 °C for 4 h, yielding yields ranging from 85 % to 97 %. It is worth noting that the catalyst showed exceptional reusability, retaining constant catalytic activity over nine cycles (Scheme 35, 36, 37).^[124]^

## 4 Conclusions

This comprehensive review has highlighted the significant advancements in developing and applying magnetic nanocatalysts for forming carbon‐sulfur (C−S) and carbon‐selenium (C−Se) bonds. The unique properties of magnetic nanocatalysts, including their high surface area, ease of recovery, and potential for recyclability, position them as highly effective tools in organic synthesis. We have discussed various synthetic strategies to create these nanocatalysts, emphasizing the importance of tailoring their composition and structure to optimize catalytic performance. The mechanistic insights from recent studies reveal the intricate pathways through which these nanocatalysts facilitate C−S and C−Se bond formation, often involving novel reaction mechanisms that differ from traditional methods. Furthermore, the diverse range of substrates successfully transformed using magnetic nanocatalysts underscores their versatility and potential for broader application in synthesizing complex organic molecules.

Despite the promising developments in this field, several challenges remain. There is a need for further exploration into the long‐term stability and activity of these nanocatalysts under various reaction conditions. Additionally, the scale‐up of these catalytic processes for industrial applications warrants further investigation to ensure their practical viability. In conclusion, integrating magnetic nanocatalysis into C−S and C−Se bond formation represents a significant stride towards more sustainable and efficient synthetic methodologies. Continued research in this area is essential for unlocking the full potential of magnetic nanocatalysts, paving the way for innovative solutions in organic synthesis and related fields. As we move forward, interdisciplinary collaboration will be crucial in addressing existing challenges and advancing the development of next‐generation catalytic systems that meet the demands of modern chemistry.

## Conflict of Interests

The authors declare no conflict of interest.

## Biographical Information

*Radwan Ali was born in Al‐Qadisiyah, Iraq. He received his BSc in Chemistry from the University of Al‐Qadisiyah, Iraq, in 2018, his MSc in Analytical Chemistry from Azad University, Mashhad, Iran, in 2020, and his PhD in Organic Chemistry from Ilam University, Iran, in 2025. His research interests center around organic synthesis and include heterocyclic synthesis, asymmetric synthesis, natural products synthesis, synthetic methodology, and applications of various catalysts in multicomponent reactions. Radwan Ali is currently an Assistant Lecturer at the University of Al‐Qadisiyah. His work involves teaching undergraduate chemistry courses, supervising laboratory sessions, and conducting research in organic chemistry. His expertise spans organic synthesis, heterocyclic compounds, magnetic nanocatalysts, and analytical techniques in chemistry*.

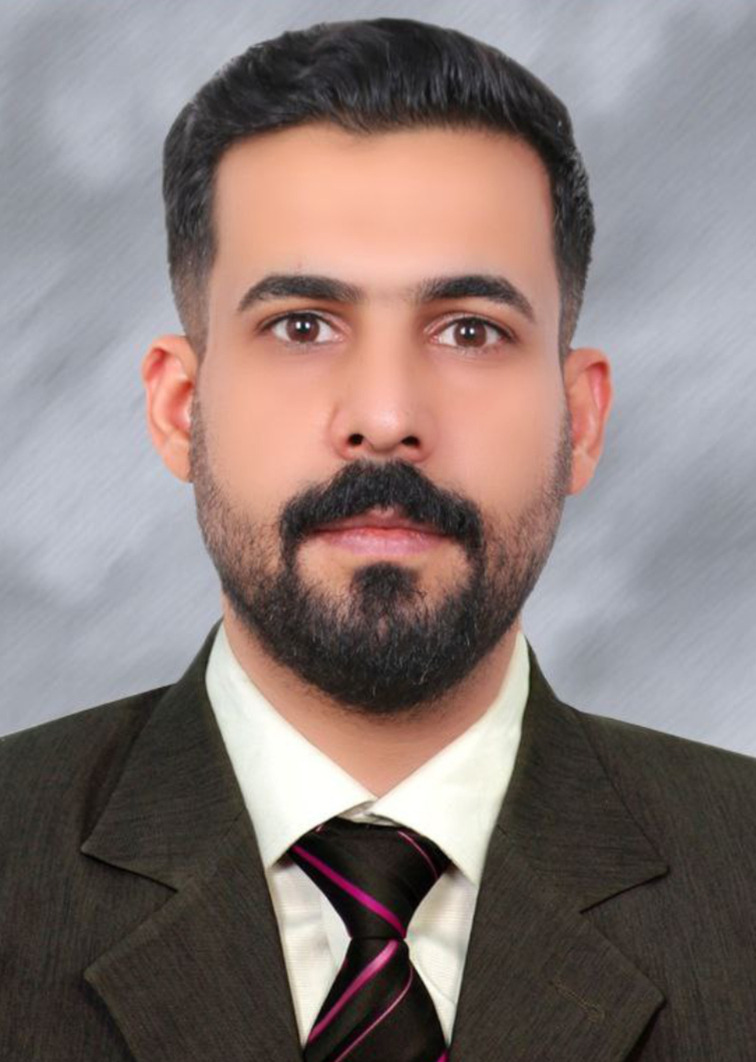

## Biographical Information

*Jianlin Han received his Ph.D. in organic Chemistry in 2007 from Nanjing University. He then carried out postdoctoral studies for one year at Texas Tech University. In 2008, he moved to the University of Oklahoma to continue postdoctoral research for nearly one year. In 2009 he took the position of Associate Professor at the Nanjing University. In 2019, he moved to Nanjing Forestry University and became a professor there. His research topics include organic fluorine chemistry, radical reaction, and asymmetric synthesis*.

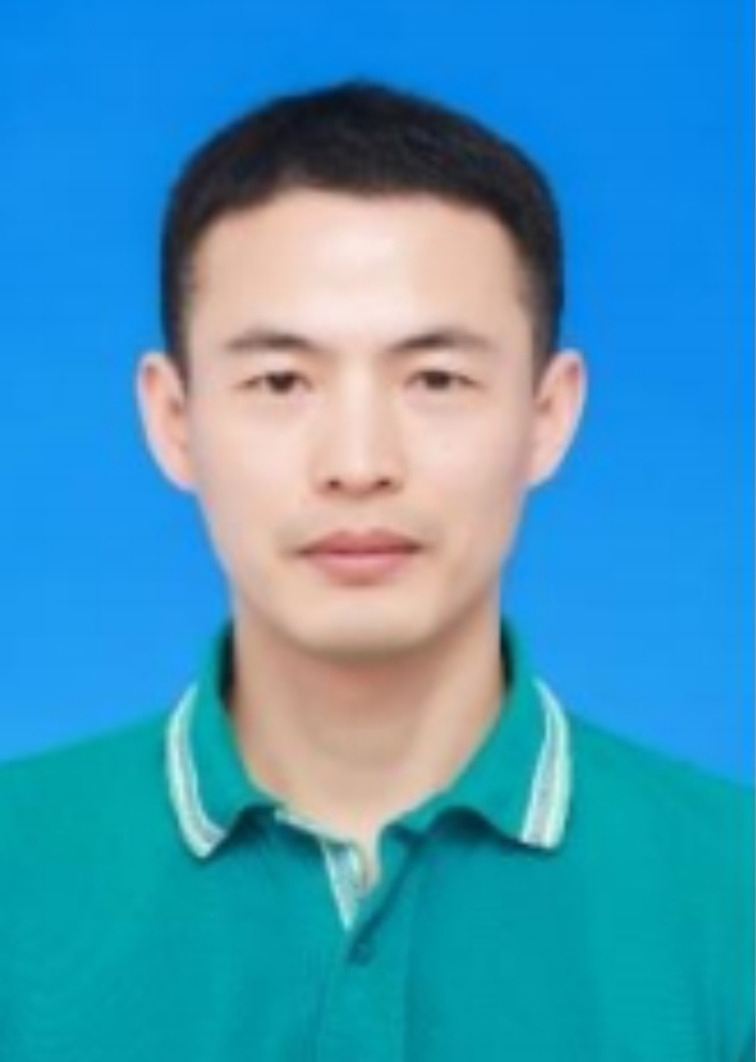

## Biographical Information

*Mosstafa Kazemi was born in Ilam, Iran. He has received MS degree in organic chemistry from Ilam University in 2013, his Ph.D. degree in organic chemistry from Ilam University in 2018. Dr. Kazemi is interested in the development of novel synthetic methods, nanocatalysts and particularly involving the application of Magnetic nanocatalysts in chemical reactions*.

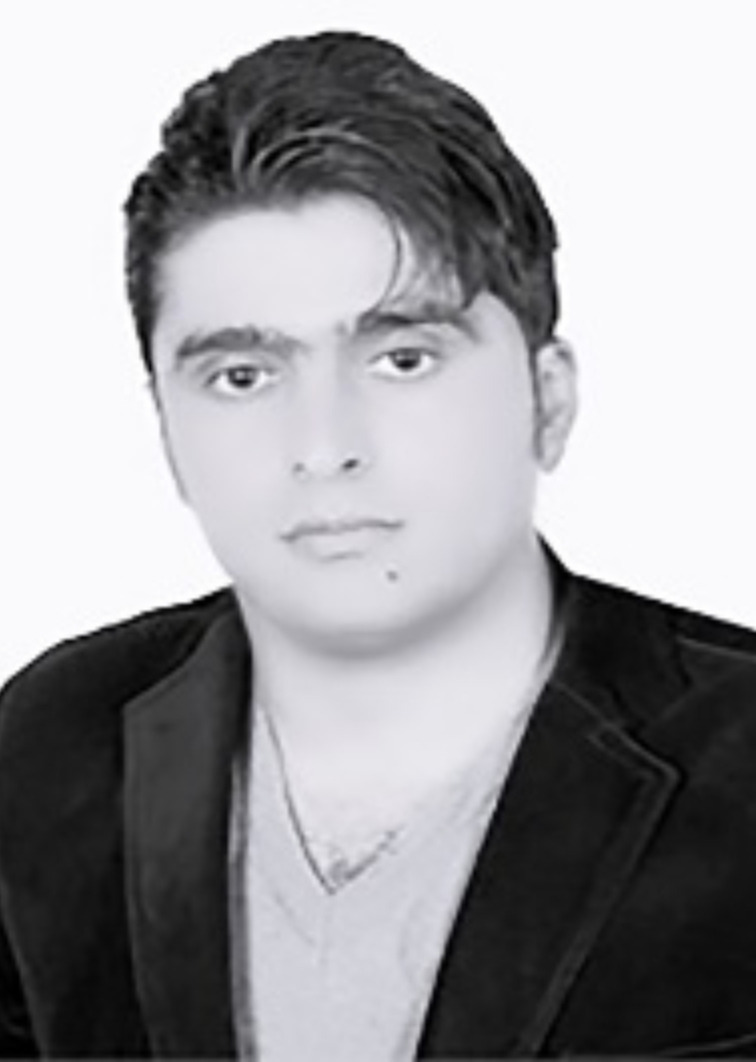

## Biographical Information

*Ramin Javahershenas was born in Urmia, Iran, in 1971. He received his BSc in applied chemistry from Tabriz University, Tabriz, Iran in 1993, his MSc in organic chemistry from Urmia University, Urmia, Iran under the supervision of Professor Naser Ardabilchi in 1999, and his PhD in organic chemistry from Urmia University, Urmia, Iran under the supervision of Professor Jabbar Khalafy in 2017. His research interests canter around organic synthesis and include heterocyclic synthesis, asymmetric synthesis, natural products synthesis, synthetic methodology, and applications of various catalysts in multicomponent reactions*.

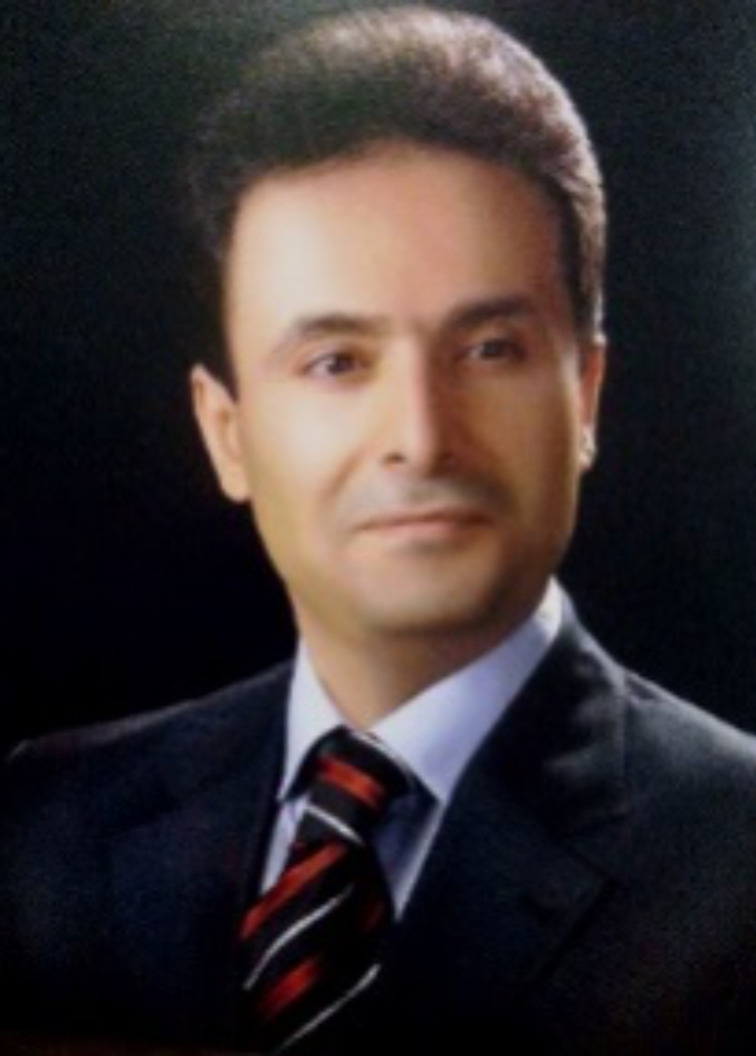

## Data Availability

The data that support the findings of this study are available from the corresponding author upon reasonable request.
